# SUN4 is a spermatid type II inner nuclear membrane protein that forms heteromeric assemblies with SUN3 and interacts with lamin B3

**DOI:** 10.1242/jcs.260155

**Published:** 2023-03-27

**Authors:** Hanna Thoma, Luisa Grünewald, Silke Braune, Elisabeth Pasch, Manfred Alsheimer

**Affiliations:** Department of Cell and Developmental Biology, Biocenter, University of Würzburg, Am Hubland, 97074 Würzburg, Germany

**Keywords:** SUN4, SUN3, LINC complex, SUN domain protein, Spermiogenesis, Nuclear envelope, Nuclear shaping, Mammals

## Abstract

SUN domain proteins are conserved proteins of the nuclear envelope and key components of the LINC complexes (for ‘linkers of the nucleoskeleton and the cytoskeleton’). Previous studies have demonstrated that the testis-specific SUN domain protein SUN4 (also known as SPAG4) is a vital player in the directed shaping of the spermatid nucleus. However, its molecular properties relating to this crucial function have remained largely unknown, and controversial data for the organization and orientation of SUN4 within the spermatid nuclear envelope have been presented so far. Here, we have re-evaluated this issue in detail and show robust evidence that SUN4 is integral to the inner nuclear membrane, sharing a classical SUN domain protein topology. The C-terminal SUN domain of SUN4 localizes to the perinuclear space, whereas the N-terminus is directed to the nucleoplasm, interacting with the spermiogenesis-specific lamin B3. We found that SUN4 forms heteromeric assemblies with SUN3 *in vivo* and regulates SUN3 expression. Together, our results contribute to a better understanding of the specific function of SUN4 at the spermatid nucleo-cytoplasmic junction and the process of sperm-head formation.

## Teaser

**Summary:** We studied the biochemical and dynamic properties of the testis-specific SUN domain protein SUN4 and provide novel insights into its interaction behavior at the spermatid nucleo-cytoplasmic junction.

## INTRODUCTION

During differentiation, most eukaryotic cells undergo fundamental changes in their function and morphology. Besides phenotypical changes of the cell itself, cellular differentiation often involves an active relocation and restructuring of the nucleus (Burke and Roux, 2009; Fridkin et al., 2009; Skinner and Johnson, 2017; Starr, 2009). One example of a very pronounced differentiation-related nuclear restructuring occurs during spermiogenesis, which describes the maturation of haploid spermatids into fertilization-competent spermatozoa. Besides the formation of the flagellum and the acrosome, spermiogenesis is primarily characterized by a prominent nuclear reshaping from round to elongated (Hermo et al., 2010, 2009; Russell et al., 1991; Kierszenbaum and Tres, 2004; Kierszenbaum et al., 2003). Nuclear elongation, in turn, is a highly organized process combining different cellular processes, including the assembly of sperm(atid)-specific cytoskeletal structures, nuclear movement, compaction of the chromatin and extensive remodeling of the nuclear envelope (NE), which comprises alterations of the general NE composition, as well as relocalization of various NE components. Best examples are the lamins B1 and B3, the lamina-associated protein LAP2 (also known as TMPO), and the lamin B receptor, which characteristically polarize to the posterior pole of the spermatid nucleus as spermiogenesis progresses (Alsheimer et al., 1998; Mylonis et al., 2004; Schütz et al., 2005a; Vester et al., 1993).

Several recent studies suggest that LINC (for linkers of the nucleoskeleton and the cytoskeleton) complexes have a key function in spermatid nuclear remodeling and elongation (Calvi et al., 2015; Gao et al., 2020; Göb et al., 2010; Pasch et al., 2015; Yang et al., 2018). LINC complexes are conserved NE-bridging assemblies that physically connect the nucleus to the cytoskeleton, thus providing a molecular basis for the transfer of mechanical forces to the NE and beyond (Crisp et al., 2006; Starr and Fridolfsson, 2010). The core of the LINC complex is formed by members of two transmembrane protein families – the Sad-1/UNC-84 (SUN) domain and the Klarsicht/ANC-1/SYNE/homology (KASH) domain proteins (Crisp et al., 2006; Razafsky and Hodzic, 2009; Starr and Fridolfsson, 2010; Stewart-Hutchinson et al., 2008). SUN domain proteins are highly conserved proteins of the inner nuclear membrane (INM) sharing a common C-terminal SUN domain (Hagan and Yanagida, 1995; Malone et al., 1999). They N-terminally anchor to structural components of the nuclear interior (e.g. nuclear lamins), whereas their C-terminal part, which consists of a central coiled-coil motif and the conserved SUN domain, locates within the perinuclear space (PNS) (Hodzic et al., 2004; Padmakumar et al., 2005). In mammals, five different SUN proteins are known – SUN1 and SUN2, which are expressed in all kinds of somatic cells (Hodzic et al., 2004; Padmakumar et al., 2005), and SUN3, SUN4 (SPAG4) and SUN5 (SPAG4L), which are testis-specific (Calvi et al., 2015; Frohnert et al., 2011; Göb et al., 2010; Pasch et al., 2015). KASH domain proteins are integral to the outer nuclear membrane (ONM) with their large N-terminal domains (NTDs) connected to the cytoskeleton and their short C-terminal KASH domains protruding into the PNS, where they specifically interact with the SUN domain of their SUN protein binding partner (Sosa et al., 2012; Starr, 2009). In their role as connectors of nuclear and cytoskeletal structures, LINC complexes are key players in various cellular processes involving nuclear movement, positioning or anchorage, cell shaping, the establishment of cell polarity, cell migration, meiotic chromosome movement, nuclear shaping and fertilization (Kracklauer et al., 2013; Razafsky and Hodzic, 2009; Starr and Fridolfsson, 2010).

Within the past decade, evidence has been accumulating that nuclear shaping and the formation of a functional sperm-head during spermiogenesis vitally depends on the cooperative function of various different LINC complex components. According to their designated functions, these components selectively localize to distinct territories of the differentiating nucleus. SUN3, for example, interacts with KASH domain protein nesprin-1 (nuclear envelope spectrin repeat protein 1); both proteins locate to the posterior NE in round and elongated spermatids and are found exclusively in regions where the manchette microtubules (MTs) contact the NE (Göb et al., 2010; Gao et al., 2020). A similar localization has been described for SUN4, which colocalizes with SUN3 at the posterior NE during the entire differentiation (Pasch et al., 2015; Calvi et al., 2015). The distribution of SUN5 remains rather ambiguous. It initially was suggested to be part of the anterior NE, there participating in acrosome biogenesis (Frohnert et al., 2011). In subsequent studies, however, it was identified as part of the posterior NE and the head-to-tail coupling apparatus (HTCA), most likely involved in regulating head-to-tail linkage (Shang et al., 2017; Yassine et al., 2015; Zhang et al., 2021). In addition to the testis-specific SUN domain proteins, SUN1 and nesprin-3 are also present in the early spermatid stages. They appear to interact with each other and transiently localize to the very posterior pole, from which they gradually disappear with advancing differentiation. Concomitantly, SUN1η, a short SUN1 isoform, appears together with nesprin-3 at the anterior nuclear region, presumably being part of the acrosomal membrane (Göb et al., 2010).

Previous studies in *Sun4* knockout mouse models have demonstrated that SUN4 is essential for correct positioning of other NE components, proper manchette assembly and, hence, for nuclear elongation and accurate sperm-head formation (Calvi et al., 2015; Pasch et al., 2015; Yang et al., 2018). A similar function was recently described for SUN3 (Gao et al., 2020). The *Sun3*-knockout phenotype in spermatids perfectly mirrors that of *Sun4*, indicating that both are involved in the same process. This is supported by the fact that correct NE localization of SUN3 and SUN4 is mutually interdependent (Calvi et al., 2015; Pasch et al., 2015; Gao et al., 2020). Based on this interdependency, it seems plausible that SUN3 and SUN4 cooperate in forming functional LINC complexes with nesprin-1 to physically link the manchette MTs to the NE.

In the case of SUN4, the particular molecular properties relating to its function are still largely unclear. According to its basic composition, SUN4 overtly resembles a typical SUN domain protein. It is expected to be integral to the INM, N-terminally connecting to the nucleoskeleton, with its C-terminal part directed to the PNS (Meinke and Schirmer, 2015). However, previous studies have suggested that SUN4 is also located at the axoneme and the HTCA. At these sites, based on evidence from *in vitro* experiments, SUN4 was assumed to bind via its SUN domain to the outer dense fiber protein ODF1 (Shao et al., 1999; Yang et al., 2012, 2018). This scenario clearly conflicts with the generally accepted bona fide membrane topology of SUN domain proteins, as the implied typical PNS localization of the SUN domain would make direct interactions with cytoskeletal elements basically impossible. To date, it has never been validated how SUN4, in reality, is oriented within the NE; its localization within the NE and its intrinsic membrane topology have remained rather hypothetical. Beyond this, it is still unclear how SUN4 is organized within a functional LINC complex and how this complex might anchor to the spermatid nucleoskeleton.

To better understand the basic function and structure of SUN4-containing LINC complexes, we conducted a detailed analysis of SUN4, intending to define its factual membrane topology and *in vivo* interaction behavior. Consistent with all previously analyzed SUN domain proteins, we here characterize SUN4 as a typical type II membrane protein that is integral to the INM, with its C-terminal SUN domain located within the PNS and its N-terminal part extending into the nucleoplasm of the spermatid nucleus. In addition, we provide evidence that of the two apparent hydrophobic motifs (HMs) identified in the SUN4 amino acid sequence, only one is a functional transmembrane domain (TMD), whereas the second is not integral but confers membrane affinity and adds to the overall membrane retention of the protein. We further show that SUN4 forms heteromeric assemblies with SUN3 in its natural context (i.e. within spermatids), and that it anchors to the nuclear lamina via a specific interaction of its NTD with lamin B3. Thus, our results provide further important insights into the specific function and behavior of SUN4 and its role in the formation of functional LINC complexes.

## RESULTS

### SUN4 is a type II transmembrane protein

**Fig. 1.**
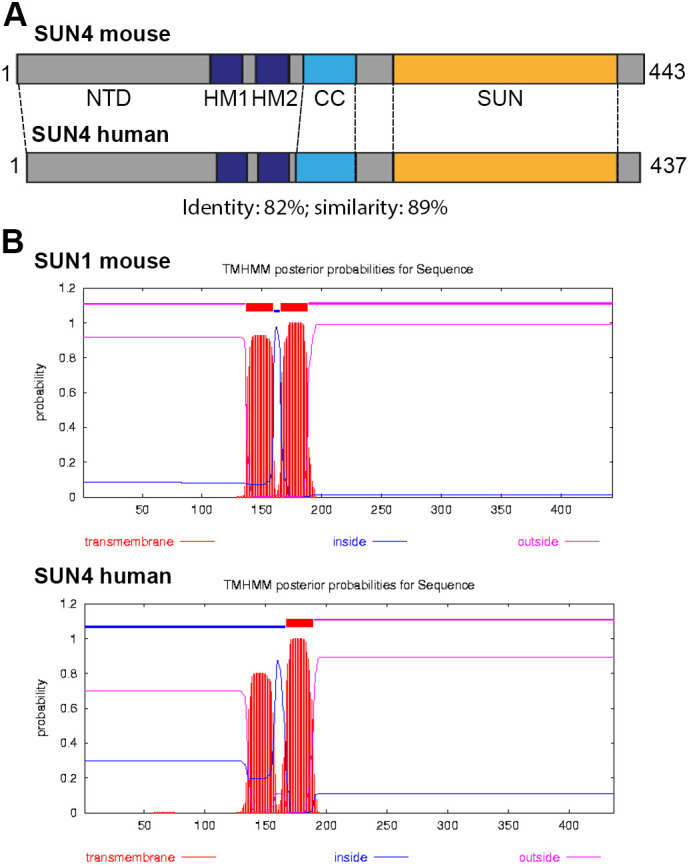
**SUN4 features two hydrophobic, potentially membrane-spanning elements.** (A) Schematic illustration of mouse and human full-length SUN4 proteins. (B) TMHMM prediction tool (http://www.cbs.dtu.dk/services/TMHMM/) identifies two hydrophobic, putative transmembrane elements, HM1 and HM2. HM1 (mouse, aa 137–159; human aa 135–157); HM2 (mouse, aa 166–188; human aa 167–189). NTD, N-terminal domain; CC, coiled-coil domain; SUN, SUN domain.

Common transmembrane prediction tools [e.g. TMHMM (https://services.healthtech.dtu.dk/) or SOSUI [http://harrier.nagahama-i-bio.ac.jp/sosui)] indicate the presence of two hydrophobic, putative membrane-spanning domains within the N-terminal half of the SUN4 protein sequence of both, mouse [HM1, amino acids (aa) 137–159; HM2, aa 166–188] and human [HM1, aa 135–157; HM2 aa 167–189] (Fig. 1). It is, however, unclear whether SUN4 is a true transmembrane protein at all. Furthermore, it has not yet been experimentally determined which of the two domains is actually membrane spanning and how SUN4 is oriented within the NE.

**Fig. 2.**
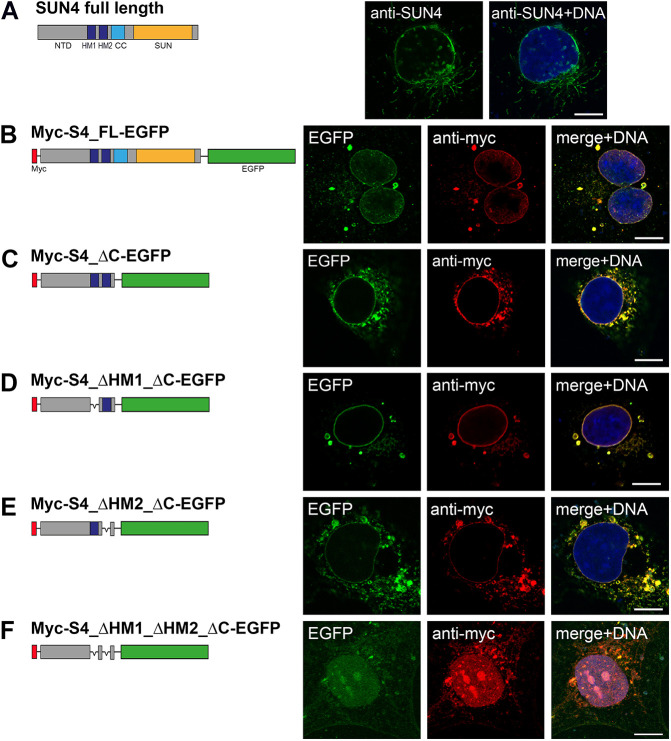
**HM1 and HM2 both confer membrane affinity to SUN4.** Wild-type mouse SUN4 and related Myc and EGFP double-tagged fusion constructs were transiently transfected into COS-7 cells and their respective expression patterns were analyzed 24 h after transfection. Confocal images of (A) SUN4 and (B–F) Myc immunostainings are shown in combination with the EGFP fluorescence. Images are representative of at least two experimental repeats. DNA was counterstained with Hoechst 33258. Scale bars: 10 µm.

To address this issue, we started with a systematic biochemical screen largely following the protocols successfully applied to define the TMDs of SUN1 and SUN5 (Frohnert et al., 2011; Liu et al., 2007). Analogous to the previous strategies, we generated different SUN4 constructs – each as Myc- and EGFP double-tagged and untagged versions – coding for either the wild-type or mutant proteins with deletions of the C-terminal domain (CTD) and either one of the putative TMDs, HM1 or HM2, or both (Fig. 2).

First, we transfected COS-7 cells with untagged wild-type or mutant SUN4 constructs and analyzed the behavior of the ectopically expressed proteins by immunofluorescence microscopy at 24 h after transfection. Consistent with what might be expected for a SUN domain protein, wild-type SUN4 displayed typical membrane affinity and localized to the NE and, likely due to ectopic overexpression, to the cytoplasmic membrane systems (Fig. 2A). Virtually the same behavior was observed with a SUN4 version flanked by an N-terminal Myc tag and a C-terminal EGFP tag (Myc–S4_FL–EGFP), suggesting that the tags do not interfere with membrane association per se (Fig. 2B). Likewise, a truncated SUN4 construct containing both HMs but lacking the C-terminal, presumably perinuclear, domain (Myc–S4_ΔC–EGFP) was still found to be associated with the endogenous membrane systems, showing a clear tendency to accumulate at the NE (Fig. 2C). Single deletions of either of the two HMs (Myc–S4_ΔHM1_ΔC–EGFP, Myc–S4_ΔHM2_ΔC–EGFP) did not prevent membrane association of the respective SUN4 constructs, indicating that each of the HMs, to some extent, confers membrane affinity. The ΔHM1 construct appeared to have higher affinity to the nuclear envelope than the ΔHM2 variant, which also showed partial NE localization. In case of ΔHM2, however, most of the protein seemed to remain in the cytoplasm, where it was associated with the endogenous membrane systems (Fig. 2D,E). Deletion of both HMs completely impeded membrane association, demonstrating that at least one of them must be present to allow efficient membrane targeting (Fig. 2F).

**Fig. 3.**
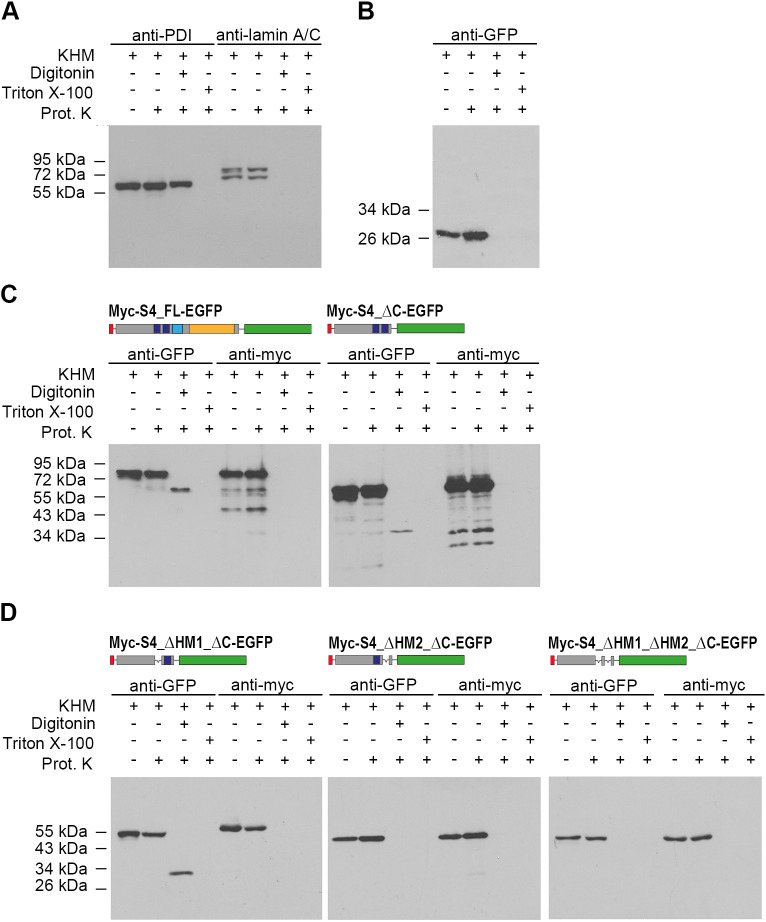
**Identification of the actual TM domain by *in situ* proteinase K digestion assay.** Untransfected Cos-7 cells (A) and Cos-7 cells transfected with pEGFP (B) or expression vectors coding for selected Myc and EGFP double-tagged SUN4 proteins (C,D) were subjected to proteinase K digestion under different membrane permeabilization conditions and processed for immunoblotting (lanes 2–4; the first lanes show controls with untreated cells). (A) Analysis of PDI and lamin A/C in untransfected cells and (B) of EGFP in pEGFP-transfected cells. (C) Cells expressing Myc and EGFP double-tagged full-length SUN4 or C-terminally truncated SUN4 were tested for proteinase digestion behavior using anti-Myc and anti-GFP antibodies. (D) Analysis of SUN4 versions with deletions of either HM1, HM2 or both. Images are representative of at least two experimental repeats. KHM, KHM buffer.

To narrow down the factual membrane-spanning domain(s) in SUN4 and define its effective membrane topology, we expressed selected SUN4 constructs and performed *in situ* proteinase K digestion assays according to established protocols (Frohnert et al., 2011; Liu et al., 2007). In brief, COS-7 cells were transfected with either of the respective Myc- and EGFP double-tagged constructs (Figs 2 and 3). At 24 h after transfection, the cells were prepared for proteinase K digestion. One set of each transfection batch was directly subjected to proteinase K digestion and a second set of cells was permeabilized with 24 µM digitonin (10 min on ice) and subsequently incubated with proteinase K. A third set was treated with proteinase K in the presence of 0.5% Triton X-100. The 0.5% Triton X-100 used in this assay permeabilizes the entire membrane system of the cell, whereas treatment with 24 µM digitonin selectively permeabilizes the plasma membrane while keeping the endogenous membrane systems intact. Thus, when treating the cells with 24 µM digitonin, protein parts located inside the membrane systems are expected to be protected from proteinase K, whereas in the presence of Triton X-100, the protease has access to all parts of the protein and therefore should be able to digest membrane proteins completely (Liu et al., 2007; Frohnert et al., 2011). Accordingly, we found ectopically expressed EGFP and the INM-associated nuclear lamins, used as a control for cytoplasmic or peripheral membrane-associated proteins, entirely degraded after permeabilization with either 0.5% Triton X-100 or 24 µM digitonin. The ER-luminal protein disulfide-isomerase (PDI), however, was degraded upon 0.5% Triton X-100 permeabilization, but was protected from protease digestion when using 24 µM digitonin (Fig. 3A,B).

First, we tested full-length SUN4 (Fig. 3C). Here, anti-Myc and anti-GFP antibodies both detected the undigested Myc- and EGFP double-tagged full-length protein (apparent molecular mass of ∼80 kDa) in untreated cells and in proteinase K-exposed cells having intact membrane systems. In contrast, in transfected cells pre-permeabilized with 24 µM digitonin, the N-terminal Myc tag signal disappeared entirely after proteinase K treatment, demonstrating that with permeabilization of the plasma membrane, the NTD became accessible for protease digestion. Under the same experimental conditions, the anti-GFP antibody still recognized a peptide that appeared significantly smaller (apparent molecular mass of ∼60 kDa), probably representing a shortened fragment lacking the NTD (∼18 kDa). Thus, after treatment with digitonin, the EGFP epitope seemed to be protected from protease digestion, suggesting that the C-terminus of SUN4 is located within the lumen of the endogenous membrane systems (Fig. 3C). As expected, proteinase K treatment after permeabilization with Triton X-100 led to degradation of the entire protein. Parallel experiments with Myc- and EGFP double-tagged SUN4 lacking the coiled-coil region and the SUN domain yielded virtually the same results as obtained with the Myc- and EGFP double-tagged full-length version, demonstrating that the C-terminal part of SUN4 has no impact on the general SUN4 topology (Fig. 3C). Together, these results provide clear evidence that SUN4 is a true type II transmembrane protein. Accordingly, its membrane topology is comparable to that of other known SUN domain proteins, with the C-terminus located within the PNS and the N-terminus protruding into the nuclear interior or the cytoplasm (Meinke and Schirmer, 2015).

### HM2 is the only valid transmembrane domain of SUN4

As a type II transmembrane protein, SUN4 requires at least one functional TMD. As mentioned above, mouse and human SUN4 protein sequences contain two hydrophobic motifs (HM1 and HM2), which, according to transmembrane prediction tools, have high probability of being membrane spanning (Fig. 1). However, according to our findings, only one can actually be a functional TMD (otherwise, the N- and C-termini would have to be situated in the same compartment). To test which of these motifs is a valid TMD, we performed proteinase K digestion assays on cells expressing mutant SUN4 proteins that lacked either HM1 or HM2 or both.

As expected, the construct lacking both HMs is completely degraded by the protease after digitonin treatment (Fig. 3D), demonstrating that at least one of the HMs is required for membrane integration (see also Fig. 2). In the case of the SUN4 construct lacking HM1 but containing HM2, the EGFP-tagged C-terminus is protected from protease digestion after digitonin permeabilization. Under the same conditions, the protein with an intact HM1 but missing HM2 was completely digested. This clearly demonstrates that, of the two putative TMDs, only HM2 is functional, whereas HM1 confers membrane affinity, as indicated by the immunofluorescence experiment (Fig. 2E) but is not integral. Thus, these experiments further substantiate that SUN4, like the other SUN protein family members, shows a typical type II membrane protein conformation consisting of a single TMD, the C-terminus located within the PNS and the NTD oriented to the nuclear interior or the cytoplasm (Meinke and Schirmer, 2015).

### The N-terminal domain of SUN4 is located within the nucleoplasm

Previous studies have described that besides its localization to the posterior NE, SUN4, to some extent, also resides at the axoneme, where its CTD is presumed to interact with ODF1 (Shao et al., 1999; Yang et al., 2012). This scenario, however, is in clear conflict with our above finding that the C-terminal part of SUN4 is protected from protease digestion in cells with intact ER-PNS membrane systems and, thus, locates in the ER-PNS lumen. This protein part should therefore not be able to directly interact with cytoskeletal proteins such as ODF1.

**Fig. 4.**
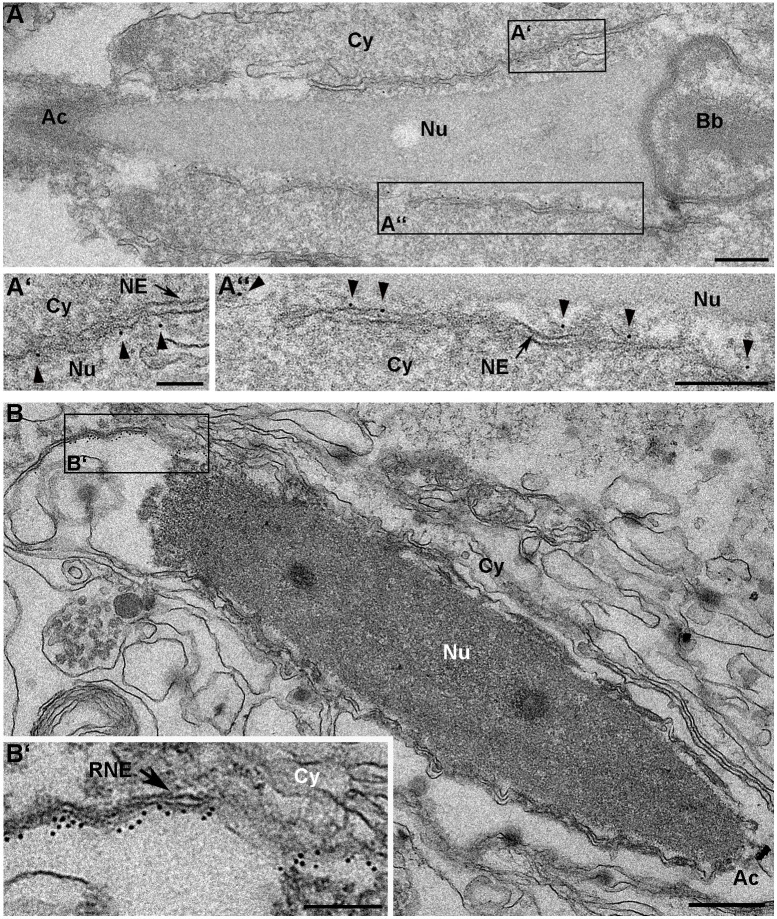
**The SUN4 N-terminal domain is located within the nucleoplasm of elongating spermatids.** Representative electron micrographs of elongating spermatids in (A) pre-fixed and (B) native mouse testis cryo-sections with immunogold labeling (6 nm colloidal gold) of the SUN4 NTD. Gold particles (arrowheads) are mainly found along the inner surface of the INM (A) and the RNE (B). Higher magnifications of the areas indicated by black rectangles are depicted in A′,A″ and B′. Scale bars: 500 nm (B); 200 nm (A,A″,B′); 100 nm (A′). Cy, cytoplasm; Nu, nucleoplasm; NE, nuclear envelope; RNE, redundant nuclear envelope; Ac, acrosome; Bb, basal body. Images are representative of two experimental repeats.

To verify the actual localization and topology of SUN4 in its natural context (i.e. the haploid spermatids), we performed immunogold EM analysis on mouse testis tissue sections, using primary antibodies against the SUN4 NTD. In elongating spermatids, we found gold particles exclusively at the nucleoplasmic surface of the INM. (Fig. 4). Consistent with our earlier immunofluorescence data (Pasch et al., 2015), the gold particles decorated the central and posterior parts of the NE, which correspond to regions covered by the manchette MTs (Fig. 4A). In late elongating spermatids, we also found considerable amounts of gold particles within the posterior chromatin-less membrane protrusions of the redundant nuclear envelope (RNE) (Fawcett and Ito, 1965; Ho, 2010; Kerr, 1991) (Fig. 4B; Fig. S1). Notably, despite using antibodies from different species and applying them on testes tissue samples fixed with different protocols, we could not detect any appreciable gold accumulations in the axoneme, as was reported in the immunogold study by Shao et al. (1999). Our approaches demonstrate that SUN4 is a protein that only localizes to the INM. This distribution is consistent with our previous finding that SUN4 exclusively locates to the posterior NE but is excluded from the fossa region and the flagellum (Pasch et al., 2015; see also Fig. 7A). In summary, our observations confirm that the NTD of SUN4 is oriented to the nuclear interior and that SUN4, also in its native environment, shares a SUN domain protein typical type II transmembrane protein conformation (Meinke and Schirmer, 2015).

### SUN4 is exceptionally mobile within the NE of somatic cells

**Fig. 5.**
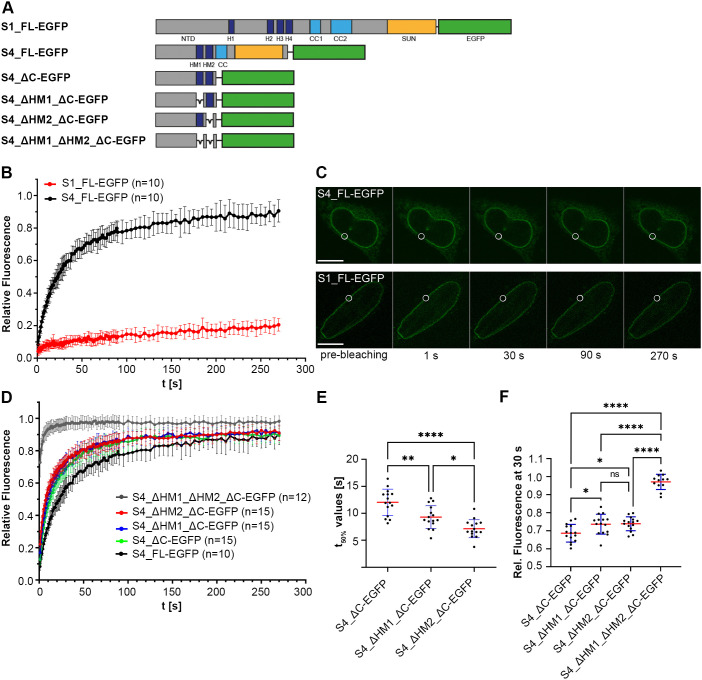
**Dynamic behavior of full-length SUN4 and selected deletion mutants expressed in NIH 3T3 cells.** (A) Schematic illustration of EGFP-tagged full-length (FL) SUN1 (with hydrophobic motifs H1–H4), SUN4 and SUN4 deletion constructs used for FRAP analyses. (B) Recovery kinetics of S1_FL–EGFP and S4_FL–EGFP. Presented values are normalized means±s.d. of 10 representative cells per construct pooled from at least five independent transfection experiments. (C) Time series of representative S1_FL–EGFP- and S4_FL–EGFP-expressing cells during FRAP analyses. Bleaching ROIs are indicated by white circles. Scale bars: 10 µm. (D) Recovery kinetics of EGFP-tagged SUN4 deletion mutants. Presented values are normalized means±s.d. of 10–15 (=*n*) representative cells per construct. (E,F) Corresponding times of 50% signal recovery (t_50%_ values) and relative signal intensities 30 s post-bleaching, respectively. Blue error bars symbolize s.d.; red middle lines represent means. ns, not significant (*P*>0.05); *P≤0.05; **P≤0.01; ***P≤0.001; ****P≤0.0001 (one-way ANOVA followed by Tukey's multiple comparisons test).

As we could demonstrate that SUN4, like the other SUN proteins, is integral to the INM, we next tested whether it shows comparable dynamic behavior within the membrane. To address this issue, we ectopically expressed EGFP-tagged full-length SUN4 (S4_FL–EGFP) in NIH 3T3 cells (see Fig. S2) and used FRAP to analyze its relative mobility compared to EGFP-tagged full-length SUN1 (S1_FL–EGFP) (Fig. 5B,C). Consistent with previous data published by Liu et al. (2007) and Hasan et al. (2006), S1_FL–EGFP was relatively immobile and showed a low recovery of only 20.5±3.9% (mean±s.d.) of the initial pre-bleach intensity after 270 s post bleach. With the same experimental setup, S4_FL–EGFP showed much higher mobility than S1_FL–EGFP. As early as 10 s post-bleaching, it recovered to ∼30% of its pre-bleach intensity, and its mean recovery rate 270 s post-bleaching even reached 90.5±6.9% (Fig. 5B). Such high recovery dynamics clearly indicate that SUN4, although effectively retained within the INM (Fig. S2; see also Fig. 2B), has a low rate of local retention, at least in a heterologous somatic environment.

### HM1 confers membrane association and affects the SUN4 dynamic behavior

As described above, HM1, although not an actual TMD, still confers membrane affinity and, when representing the only hydrophobic motif present, efficiently targets SUN4 to the endogenous membrane system (Fig. 2E). Therefore, we next aimed to identify the particular impact of HM1 on the overall membrane behavior of SUN4. In this regard, we generated another set of SUN4 deletion constructs containing one, both or none of the HMs (see Fig. 5A; Fig. S2) and studied their dynamic properties within the NE *in vivo*. To get an impression of the specific impact of HM1 and HM2, and to exclude undesirable side effects that might arise from the luminal coiled-coil and SUN domains, we employed EGFP-tagged C-terminally truncated versions of the SUN4 full-length protein and analyzed their dynamic properties by FRAP. Predictably, as shown in Fig. 5D, the C-terminal truncation led to a general overall increase in mobility compared to the full-length protein, which most likely can be ascribed to the disrupted coiled-coil and/or SUN domain interactions.

To compare the different deletion constructs with each other, we chose two distinct reference values: (1) the averaged time points when the respective constructs had regained 50% of their pre-bleach intensity (t_50%_ values), and (2) their relative intensity at 30 s post-bleaching (Fig. 5E; a list with the detailed *P*-values can be found in Table S1). As shown in Fig. 5D–F, the construct containing both HMs (S4_ΔC–EGFP), featured the lowest mobility of the four constructs, with a t_50%_ value of 12.0±2.4 s (mean±s.d.) and 68.6±4.9% signal recovery 30 s post-bleaching. The two constructs lacking either HM2 (S4_ΔHM2_ΔC–EGFP) or HM1 (S4_ΔHM1_ΔC–EGFP) both displayed significantly increased mobility compared to S4_ΔC–EGFP. S4_ΔHM1_ΔC–EGFP showed a t_50%_ value of 9.3±2.1 s and reached 73.6±5.5% of its pre-bleach intensity after 30 s. S4_ΔHM2_ΔC-EGFP showed a quite similar degree of mobility with a t_50%_ value of 7.1±1.7 s, and 73.8±3.8% recovery after 30 s; the t_50%_ value appeared to be only slightly lower than that of S4_ΔHM1_ΔC-EGFP (see Fig. 5E,F). The S4_ΔHM1_ΔHM2_ΔC–EGFP mutant lacking both HMs showed by far the highest mobility, reaching 72.7±9.0% of the initial signal intensity 1 s post-bleaching and nearly full recovery (97.1±4.2%) within the first 30 s. This, in other words means, (1) when HM1 is the only HM present in SUN4_ΔC, it reduces the mobility of the protein to a similar extent to that observed when only HM2, the true TMD, is present; and (2) when the protein is actually integrated into the membrane (i.e. it contains HM2), the additional presence of HM1 provokes a weak, but discernible additional decrease in mobility (Fig. 5E,F). From this, we conclude that HM1, although not membrane spanning, conveys NE targeting and – similar to the N-terminal HMs in SUN1 (Liu et al., 2007) – adds to the overall membrane retention of SUN4.

### SUN4 forms heteromeric assemblies with SUN3

Because full-length SUN4 appeared rather mobile when ectopically expressed in NIH 3T3 cells, we hypothesized that in its natural context (i.e. in spermatids), SUN4 stabilization and retention within the NE depends on spermatid-specific partners.

Previous studies have demonstrated that in round and elongating spermatids, SUN4 localizes to the regions where the MT manchette is in tight association with the NE (Calvi et al., 2015; Pasch et al., 2015). Notably, at these sites, SUN4 distributes congruently with SUN3. In addition, in previous *in vitro* experiments, SUN3 co-immunoprecipitated together with SUN4, indicating their intrinsic capacity to interact with each other (Pasch et al., 2015). Therefore, it seems conceivable that, in their natural environment, SUN4 and SUN3 are interacting partners forming joint LINC complexes, which in this combination are functional in a higher-order spermatid-specific nucleocytoplasmic network system.

To prove this hypothesis, we tested whether SUN4 indeed forms complexes with SUN3 in spermatids. In this regard, we performed co-immunoprecipitation (co-IP) from testicular cell suspensions of wild-type mice, using a protocol optimized for nuclear membrane proteins. In a first step, we employed 8 M urea extraction (see Lang et al., 1999), followed by high-speed centrifugation (100,000 ***g***) to selectively isolate the cellular membranes together with internal membrane proteins. Subsequently, the membranes were dissolved in radioimmunoprecipitation assay (RIPA) buffer and subjected to classical co-immunoprecipitation using anti-SUN4 antibodies directed against the NTD of SUN4. In parallel, we used non-specific IgGs as a negative control. Immunoprecipitates and corresponding supernatants were then probed with anti-SUN3 antibodies.

**Fig. 6.**
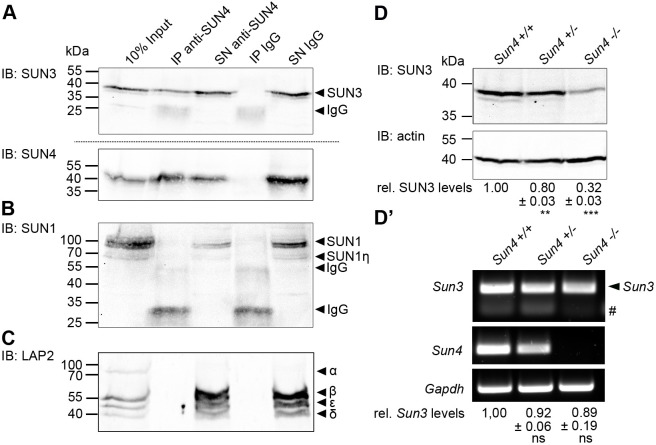
**SUN4 forms heteromeric assemblies with SUN3 in mouse spermatids and SUN3 protein levels are decreased in SUN4 knockout tissue.** (A–C) Western blots of rabbit anti-SUN4 immunoprecipitated membrane protein extracts from mouse testis cells. Non-specific rabbit IgGs were used as a negative control. Precipitates (IP) and supernatants containing unbound proteins (SN) were probed with (A) guinea pig anti-SUN3 and anti-SUN4, (B) anti-SUN1 and (C) mouse anti-LAP2 antibody (immunoblot, IB). Untreated testis cells served as input control. LAP2 isoforms were assigned according to Berger et al. (1996). Please note that LAP2α does not contain a TMD and thus can not be present in the SN. Amounts of loaded cell equivalents: 10% input, 1.5×10^6^; IP, 1.5×10^7^; SN, 1.5×10^7^. Images in A–C are representative of at least two experimental repeats. (D) Immunoblot detection of SUN3 levels in *Sun4^+/+^*, *Sun4^+/−^* and *Sun4^−/−^* whole-testis lysates; actin protein levels served as loading control. Relative SUN3 protein expression levels normalized to actin (±s.d. from three independent experiments) are given below. (D′) Semi-quantitative RT-PCR analysis of *Sun3* mRNA levels in *Sun4*^+/+^, *Sun4*^+/−^ and *Sun4*^−/−^ mouse testis cells. *Sun4* and *Gapdh* mRNA levels were monitored as control. Relative *Sun3* mRNA levels normalized to *Gapdh* (±s.d. from three independent experiments) are shown below. Hash symbol indicates background signals of the primers. Two-tailed one-sample *t*-tests were performed to valuate the significance of the differences between *Sun3* mRNA and SUN3 protein levels in Sun4*^+/+^*, *Sun4^+/−^* and *Sun4^−/−^* tissues. ns, not significant (*P*>0.05); *P≤0.05; **P≤0.01; ***P≤0.001.

As expected, SUN4 was efficiently precipitated with the anti-SUN4 antibodies, but was not detected in the control precipitate (Fig. 6A). Consistent with our hypothesis, SUN3 was also found in high amounts in the anti-SUN4 precipitate but not in the IgG control. This demonstrates that SUN4 and SUN3 indeed interact with each other and form heteromeric assemblies not only *in vitro* but also in their natural background (i.e. in spermatids).

### SUN4 is required for maintenance of SUN3 protein levels

A recent study has shown that in spermatids, SUN3 considerably affects the expression of SUN4, i.e. SUN4 protein levels are decreased in the absence of SUN3 (Gao et al., 2020). Therefore, we asked whether this is also true vice versa, namely, whether SUN4 has a similar effect on SUN3 expression. To explore this, we studied SUN3 protein expression in whole-testis lysates of *Sun4^+/+^*, *Sun4^+/−^* and *Sun4^−/−^* mice by immunoblot analysis. As shown in Fig. 6D, in the *Sun4^−/−^* background, the SUN3 amount appeared to be significantly reduced compared to the wild-type (32±3%; mean±s.d. *n*=3; *P*<0.001) and heterozygous tissues, whereas the levels of actin, which were monitored as a control, remained comparable between all genotypes. We next asked whether this also applies for *Sun3* mRNA expression and therefore studied the *Sun3* transcript levels in *Sun4^+/+^*, *Sun4^+/−^* and *Sun4^−/−^* background. To this end, we performed RT-PCR on total RNA of testicular cell suspensions from mice of the respective genotypes. Contrary to the SUN3 protein levels, the *Sun3* mRNA levels in *Sun4*^−/−^ mice did not change significantly compared to those in the wild-type (89±19%; mean±s.d.; *n*=3; *P*>0.40; see Fig. 6D′). These findings clearly demonstrate that SUN4 is indeed essential for the maintenance of SUN3 protein levels. *Sun3* transcription, however, appears not to be significantly affected by the absence of SUN4. Taken together, our results suggest that SUN3 and SUN4, at least at the protein level, not only interact structurally but also appear to be regulatory linked.

### SUN4 interacts with lamin B3 in mouse spermatids

In a previous study (Pasch et al., 2015), we demonstrated that depletion of SUN4 not only results in mislocalization of its LINC complex partners but also interferes with general NE integrity and leads to severe malformation of spermatids. Hence, it appears plausible that SUN4, directly or indirectly, is interconnected with other components of the NE, forming a nucleo-cytoplasmic interactome that, as a whole, orchestrates sperm-head formation.

To explore whether SUN4 interacts with other constituents of the spermatid INM showing comparable posterior localization (i.e. SUN1 and LAP2), we probed SUN4 co-IP samples with antibodies against these two proteins. As shown in Fig. 6B,C, neither SUN1 nor LAP2 (or any of their isoforms) were detectable in the anti-SUN4 precipitate, indicating that they might not effectively bind to SUN4 or SUN4-containing complexes. On the other hand, the absence of SUN1 in the anti-SUN4 precipitate substantiates that the detected SUN4–SUN3 interaction described above is specific and not a result of a general intrinsic heteromerization tendency of SUN domain proteins, which could force interactions among one another *in vitro*.

**Fig. 7.**
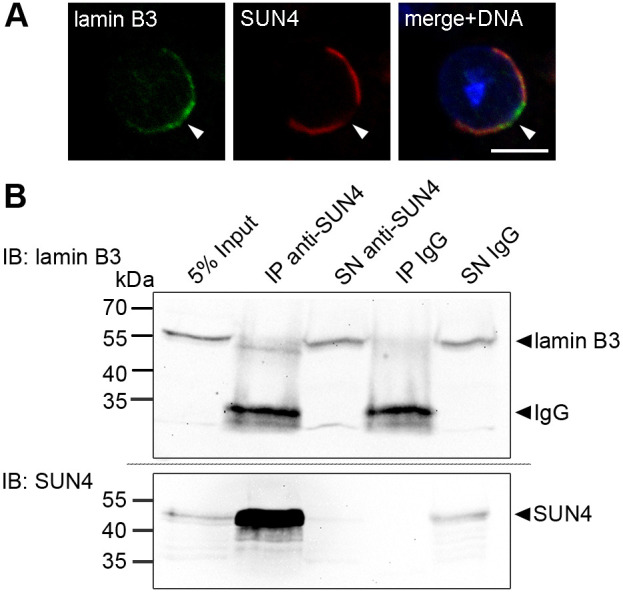
**SUN4 binds to lamin B3 in mouse spermatids.** (A) Confocal microscopy image of an elongating spermatid co-immunolabeled with anti-lamin B3 (green) and anti-SUN4 antibodies (red). DNA was counterstained with Hoechst 33258 (blue). Scale bar: 5 µm. Arrowheads indicate the implantation fossa. (B) Immunoblot (IB) is of co-immunoprecipitation from mouse testicular suspension cells with guinea pig anti-SUN4 NTD antibody and non-specific guinea pig IgG as a negative control. Precipitates (IP) and supernatants containing unbound proteins (SN) were probed with rabbit anti-lamin B3 and anti-SUN4 peptide antibodies. Untreated testis cells served as input control. Loaded cell equivalents: 5% input, 2×10^6^; IP, 4×10^7^; SN, 5×10^6^. Note, the SUN4 immunoblot was reused in Fig. S3 as the same co-IP samples were used to test for SUN4-lamin B1 interaction (Fig. S3). Images in A and B are representative of at least two experimental repeats.

**Fig. 8.**
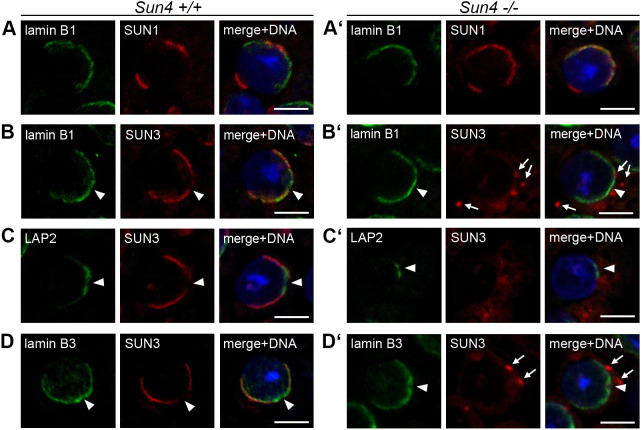
**SUN4 deficiency affects SUN1, but not lamin B3, lamin B1 or LAP2 distribution.** Testis paraffin sections of adult *Sun4^+/+^* and *Sun4^−/−^* mice were co-immunofluorescence labeled with antibodies against (A,A′) lamin B1 (green) and SUN1 or (B,B′) SUN3 (red), (C,C′) LAP2 (green) and SUN3 (red) and (D,D′) lamin B3 (green) and SUN3 (red). DNA was stained with Hoechst 33258 (blue). Shown are images of round and early elongating spermatids. Scale bars: 5 µm. Arrowheads point at the implantation fossa, arrows at cytoplasmic SUN3 aggregates typically found in *Sun4^−/−^* spermatids. Images are representative of at least two experimental repeats.

Two other proteins that show a posterior localization during nuclear elongation are the peripheral NE proteins lamin B1 and lamin B3, the only two lamin isoforms expressed in spermatids (Schütz et al., 2005a; Vester et al., 1993). As described for SUN1 and LAP2, the distribution of the two lamins partially overlaps with that of SUN3 and SUN4 (Figs 7A and 8B,C).

Given that SUN domain proteins are supposed to directly connect to the nuclear lamina (Starr and Fridolfsson, 2010), lamins B1 and B3 are prime candidates for a direct interaction with SUN4. To test whether SUN4 binds to one or both of them *in vivo*, we again performed SUN4 co-IP from testicular cells of wild-type mice. However, because lamin B1 and lamin B3 are nucleoplasmic proteins, we followed standard IP procedures and directly subjected the cells to RIPA lysis. The resulting precipitates and supernatants were probed with both anti-lamin B3 and anti-lamin B1 antibodies.

Using this assay, lamin B1 was not detectable in either the control lysate precipitated with non-specific IgG or the anti-SUN4 precipitate (Fig. S3), suggesting that it is not be a direct interaction partner of SUN4. Lamin B3, however, indeed was detectable in the anti-SUN4 precipitate but not in the IgG control (Fig. 7B). This result clearly supports the notion that SUN4 directly or indirectly interacts with lamin B3 in spermatids.

### SUN4 affects the distribution of SUN3 but not of the NE components lamin B3, LAP2 or lamin B1

In our previous study, we demonstrated that SUN4 depletion results in a severe mislocalization of its putative LINC complex partners SUN3 and nesprin-1. Moreover, in SUN4 deficient spermatids, we found a significantly altered distribution of SUN1 and nesprin-3 compared to that seen in wild-type cells (Pasch et al., 2015, see also Fig. 8A,A′).

To explore to what extent depletion of SUN4 affects the localization of other NE components not yet analyzed, we performed further co-immunofluorescence analysis on *Sun4^+/+^* and *Sun4^−/−^* testis tissues and tested for SUN3 distribution and, particularly, for LAP2, lamin B1 and B3.

Consistent with earlier studies (Alsheimer et al., 1998; Göb et al., 2010; Vester et al., 1993), in a wild-type background, we found that lamin B1 and LAP2 in round spermatids are localized at the posterior nuclear periphery, with a local concentration at the most posterior area neighboring the implantation fossa, and laterally overlapping with SUN3 (Fig. 8B,C). With progressing elongation, both proteins gradually retreated towards the posterior pole, a region where SUN3 is completely absent (Alsheimer et al., 1998; Göb et al., 2010). On depletion of SUN4, SUN3 entirely disappeared from the nuclear periphery and accumulated outside the nucleus in spot-like aggregates (Fig. 8B′–D′; Pasch et al., 2015). Strikingly, absence of SUN4 did not cause any visible changes in the distribution of lamin B1 or LAP2. In the SUN4-deficient background, lamin B1 and LAP2 still localized to the posterior NE. In contrast to the situation for SUN1, which in *Sun4^−/−^* spermatids caudally extends to the more lateral regions (Fig. 8A′; Pasch et al., 2015), their confinement to the posterior pole was apparently not affected either (Fig. 8B′,C′).

In wild-type spermatids, lamin B3, which we identified as a bona fide SUN4 interaction partner (see above), also localized to the posterior NE (Fig. 8D; Schütz et al., 2005a). In addition, and consistent with the previous findings, a significant proportion localized to the nucleoplasm (Fig. 8D; Schütz et al., 2005a). Remarkably, in the *Sun4^−/−^* tissues, we were not able to detect any overt changes in either the nucleoplasmic or the NE-associated pool, which showed a clear concentration around the fossa region (Fig. 8D′). Thus, although directly or indirectly interconnected with SUN4, the localization of lamin B3 seems to be independent of SUN4.

## DISCUSSION

Nuclear elongation and reorganization are key elements of male germ cell differentiation. Any defects in these processes usually lead to defective sperm function and cause severe fertility problems, often resulting in total male infertility (Yan, 2009). The exact mechanisms and networks underlying these shaping and restructuring events, however, are still poorly understood. According to current theories, sperm-head formation and nuclear elongation are guided by mechanical forces, which are supposed to be generated by highly organized cytoskeletal structures, namely, the acroplaxome and the MT manchette (reviewed by Dunleavy et al., 2019 and Teves and Roldan, 2022).

In their role as nucleo-cytoskeletal linkers, LINC complexes represent perfect candidates for physically connecting these structures to the nucleus, thus enabling a transfer of cytoskeletal shaping forces (Dunleavy et al., 2019; Kmonickova et al., 2020; Kracklauer et al., 2010; Teves and Roldan, 2022). Consistent with this, previous studies have identified the spermiogenesis-specific LINC complex components SUN4 and SUN3 as crucial players in sperm-head formation that are essential for proper NE attachment of the manchette MTs, correct positioning of other LINC complex components and finally also for directed nuclear elongation (Calvi et al., 2015; Gao et al., 2020; Pasch et al., 2015; Yang et al., 2018).

Although important for understanding its central function, the intrinsic molecular properties of SUN4 and its specific LINC complex-dependent and/or -independent interplay with other components of the spermatid NE remained largely unknown. In particular, it has remained unclear how SUN4 is oriented within the NE and how it is organized within a functional LINC complex. In the present study, we have tackled several of these questions in detail.

### SUN4 shares the typical membrane topology of a SUN domain protein

Even though it has the typical molecular composition of a SUN domain protein, the actual localization of SUN4 and its orientation within the NE were not entirely clear. Several previous studies have stated scenarios for SUN4 that are in apparent conflict with the classical topology of SUN domain proteins (Shao et al., 1999; Tapia Contreras and Hoyer-Fender, 2021; Yang et al., 2012, 2018). Based on SUN4 immunogold labeling, Shao et al. (1999) showed that, besides its currently accepted localization to the NE regions that are decorated by the manchette (Calvi et al., 2015; Gao et al., 2020; Pasch et al., 2015), SUN4 appears to be also located within the axoneme. Using an *in vitro* assay, they found indications that within the axoneme, SUN4 might bind via a C-terminal leucine zipper motif to the cytoskeletal protein ODF1 (Shao et al., 1999). This, however, is difficult to reconcile with a classical SUN domain protein topology, as such an interaction would implicate an atypical cytoplasmic orientation of the C-terminal SUN domain rather than a regular SUN domain localization within the PNS (Crisp et al., 2006). Interestingly, bioinformatical tools predicted two hydrophobic, potentially transmembrane motifs within the SUN4 amino acid sequence, offering various possibilities of how SUN4 could be oriented within the NE – including those in which the SUN domain could actually locate to the cytoplasm. In a scenario when the SUN domain of SUN4 indeed faces the cytoplasm, it might, hypothetically, then also directly bind to ODF1. Our present experiments, however, definitely rule out this option. Our systematic biochemical screening, combined with immunogold labeling of the SUN4 NTD in spermatids, identified HM2 as the only true TMD. Beyond this, we provide unequivocal evidence that the C-terminal part of SUN4 locates within the PNS or ER whereas the NTD, in spermatids, is directed to the nuclear interior. Thus, we have now demonstrated that SUN4 actually has the typical SUN domain protein membrane topology. Moreover, we now can rule out any direct interactions between the SUN4 CTD and cytoplasmatic proteins. Also, we were unable to detect the previously described axonemal localization of SUN4 (Shao et al., 1999). Although using antibodies from different species and applying them on variably fixed testis tissues, we could not reproduce any gold labeling of the axoneme.

Instead, we could validate our previous observation that SUN4 exclusively localizes to the NE, where it is particularly enriched at the lateral posterior regions and the INM of the RNE (Pasch et al., 2015; Fig. 4; Fig. S1). It is noteworthy that Yang et al. identified both ODF1 and SUN4 as essential components for proper head-to-tail coupling (Yang et al., 2012, 2018). Considering these observations, but also taking into account our results that SUN4 is not present in the axoneme, we suggest that instead of operating as interacting binding partners at the axoneme, SUN4 and ODF1 could rather be part of a joint complex located at the rim of the basal plate, where they might have a yet largely unknown function in tethering the sperm tail to the NE.

Our immunofluorescence and FRAP analyses suggest that the second hydrophobic element HM1, although not a TMD, contributes to the general membrane targeting and NE retention of SUN4. A similar phenomenon was described for SUN1, which in total contains four HMs. At least one of them is not an actual TMD but responsible for local membrane retention of the protein (Liu et al., 2007; Majumder et al., 2018). How exactly this additional retention is mediated remained largely unclear. Liu et al. (2007) suggested two possible scenarios that both, theoretically, might also apply for SUN4 – (1) that the additional HMs could function as binding sites for other INM integrated proteins, or (2) they could interact directly with the INM lipid bilayer. In this context, a mechanism similar to that of nuclear lamins would be conceivable. Lamins are targeted and tethered to the INM by adding extra hydrophobic elements through post-translational farnesylation of the CaaX box motif (Kitten and Nigg, 1991; for a review, see Perez-Sala, 2007). However, differing from the lamins, in the case of the SUN proteins, membrane targeting would here be mediated by a short peptide motif instead of an isoprene annex.

### SUN4 and SUN3 are mandatory LINC complex partners

In our FRAP analyses, we found that in a somatic context, compared to SUN1, SUN4 shows exceptional mobility within the NE. Hence, SUN4 overtly requires further spermatid-specific binding partners for effective NE retention. SUN3 would be a good candidate for being one of these retention factors, as it is congruently distributed, shares the capacity to bind nesprin-1 (Göb et al., 2010; Pasch et al., 2015) and, as shown in our study, specifically co-precipitates with SUN4 from mouse spermatids. Interestingly, Gao and colleagues recently demonstrated that, vice versa, the SUN4 protein co-precipitates with endogenous SUN3 (Gao et al., 2020). In their study, they further demonstrated that NE localization of SUN4 implicitly depends on the presence of SUN3, a phenomenon that we described several years before for SUN3 in a SUN4-deficient background. Upon SUN4 deficiency, SUN3 completely disappears from the spermatid NE and, vice versa, when SUN3 is absent, SUN4 is not recruited to the NE but is diffusely distributed in the cytoplasm (Pasch et al., 2015; Calvi et al., 2015; Gao et al., 2020). Both, the *Sun3* and *Sun4* knockout mouse models thus have provided proof for a strict interdependency of SUN3 and SUN4 regarding their localization. They point to a coordinated function, either as parallel or as joint LINC complex assemblies that might be formed based on direct or indirect interactions between SUN3 and SUN4. Remarkably, the NTD of SUN3 is pretty short and comprises only seven amino acids (Göb et al., 2010). Therefore, it appears rather unlikely that SUN3 can effectively anchor to nuclear structures. Thus, SUN3 INM retention and correct NE localization somehow need support, for example, by binding to SUN4, which in turn connects to the nuclear lamina. We therefore propose that SUN3 and SUN4 form joint LINC complexes or LINC complex systems, in which SUN3 stabilizes the connection to their putative KASH binding partner nesprin-1 and SUN4 provides the interconnection to the nuclear lamina.

The actual organization of such SUN3–SUN4–nesprin-1 LINC complexes, however, remains obscure. According to the current model, which is mainly based on structural analysis of the human SUN2–KASH1 and SUN2–KASH2 complexes, SUN proteins form trimers that connect to three KASH peptides (Sosa et al., 2012). On the other hand, Hennen and colleagues found that SUN1 and SUN3 have the potential to form even higher oligomeric assemblies (Hennen et al., 2018; Hennen et al., 2019). It is unclear which of these scenarios applies for SUN3 and SUN4 in spermatids and whether they form heteromeric coiled-coil trimers or oligomers or arrange into higher-order LINC complex systems as homomers. Structured illumination microscopy along with biochemical studies, however, indicate that SUN3 and SUN4 might actually occur as homomers, but to some extent do also form joint heteromeric assemblies in the spermatid INM (Pasch et al., 2015).

Interestingly, besides the overt above-described functional interdependency within the nucleo-cytoplasmic network system, SUN3 and SUN4 also seem to hinge on each other on a regulatory level. Recently, Gao et al. (2020) have shown that compared to what is seen in the wild type, the levels of SUN4 are significantly reduced in SUN3-depleted testis tissue. In our current study, we have now demonstrated that, conversely, the SUN3 protein levels are also significantly reduced in a *Sun4^−/−^* background. Apparently, both proteins require the presence of the other to keep up their levels. Interestingly, the *Sun3* mRNA levels are very similar between the different *Sun4* genotypes. Thus, SUN3 appears not to be regulated at the transcriptional level but rather post-transcriptionally. Given this, it is conceivable that either the *Sun4* mRNA and/or the SUN4 protein function as positive regulator of the *Sun3* mRNA translation. On the other hand, in the absence of SUN4, SUN3 might undergo proteasomal degradation, exocytosis or be preferentially directed to the residual body for elimination. As SUN3 is severely mislocalized in the *Sun4*^−/−^ background and thus apparently non-functional without SUN4, one could speculate that post-transcriptional reduction of SUN3 might be an attempt to prevent its cytoplasmic accumulation and aggregation in order to preserve general membrane integrity. However, further studies are needed in the future to enlighten this phenomenon and whether this also applies to SUN4 downregulation in the SUN3-deficient background.

### Lamin B3 represents a bona fide nucleoskeletal binding partner of SUN4 containing LINC complexes

LINC complexes are classically defined as linkers of the nucleoskeleton and the cytoskeleton (Crisp et al., 2006). As evidenced by *Sun3* and *Sun4* knockout models, and corroborated by immunolocalization, SUN3- and SUN4-containing LINC complexes connect to the MT manchette at the cytoplasmic surface of the NE (Calvi et al., 2015; Gao et al., 2020; Pasch et al., 2015). Respective nucleoskeletal counterparts, however, were not known so far. Somatic SUN domain proteins directly connect to the nuclear lamina with their NTDs (Starr and Fridolfsson, 2010). Given that spermatids also form a lamina, although considerably modified regarding the basic composition and local distribution (Alsheimer et al., 1998; Schütz et al., 2005a; Vester et al., 1993), a similar scenario is conceivable for LINC complexes in spermatids. In spermatids, only two lamins, lamin B1 and lamin B3, are present and thus represent the only possible lamin interaction partners for SUN3 and SUN4 (Schütz et al., 2005a; Vester et al., 1993). In the case of SUN3, owing to its very short NTD a direct interaction with the lamina appears pretty unlikely (see above). The NTD of SUN4, however, consists of 165 amino acids (Fig. 1), which are directed to the nuclear interior (Fig. 4) and therefore are perfect target for a direct interaction with nuclear lamins.

Lamin B1 and B3 both localize to the posterior half of the nucleus in round spermatids, largely colocalizing with SUN3 and SUN4 (Figs 7A and 8B). With progressing differentiation, a proportion of the two lamin pools congregate at the very posterior pole, where SUN3 and SUN4 are absent, and only the lateral, NE-associated portions remain as potential binding targets for SUN3 and SUN4. Of note, in the case of lamin B3, besides the NE-associated pool, a significant proportion is distributed within the nucleoplasm, having no tight contact with the nuclear periphery and, hence, cannot be the primary target for interaction with LINC complexes (Schütz et al., 2005a).

Yeh et al. (2015) found that human lamin B1 *in vitro* co-precipitates with ectopically co-expressed SEPT12 and SUN4 and that, in this artificial context, these three proteins together overtly form higher-order complexes. We, however, could not detect any hints for a direct or indirect interaction between SUN4 and lamin B1 in its natural context (i.e. spermatids). It has, admittedly, to be noted that lamin B1 usually forms stable, salt-resistant polymer networks (Schirmer and Gerace, 2004). Thus, with the RIPA buffer system, only a marginal portion of lamin B1 could be solubilized in our *in vivo* co-IP assay. Such a small amount of soluble lamin B1 might be too limited to detect true SUN4–lamin B1 complexes. Therefore, we currently cannot rule out that there is indeed no direct interaction between the SUN4 NTD and lamin B1 in spermatids.

Nonetheless, we identified lamin B3 as a true interaction partner of SUN4. Considering the differences in its local distribution compared to that of SUN3 and SUN4, we assume that, of the entire lamin B3 pool, only the fraction associated with the lateral NE regions can participate in such an interaction. In contrast to what is seen for other B-type lamins, lamin B3 is highly dynamic, shuttling between the nucleoplasmic and peripheral pool (Schütz et al., 2005b). Lamin B3 is assumed to support a flexibilization of the lamina network system in spermatids, required to facilitate the restructuring of the spermatid nucleus (Schütz et al., 2005b; Kracklauer et al., 2013). In the context of the striking, orchestrated NE dynamics observed in spermatids, crucial for nuclear shaping (for a review, see Kracklauer et al., 2013), the specific interaction between lamin B3 and SUN4 might hold a regulative function (1) in transforming the cytoplasmic shaping forces to the nuclear interior and/or (2) in supporting local NE flexibilization by directing lamin B3 to the posterior NE, where it might weaken the basic rigid lamina scaffolding provided by lamin B1. A comparable function was postulated for meiosis-specific lamin C2 in telomere-driven chromosome movement during meiotic prophase I (Link et al., 2013).

Surprisingly, absence of SUN4 in spermatids virtually did not interfere with the general lamin B3 distribution, indicating that the specific localization of lamin B3 to the lateral and posterior NE is not exclusively dependent on SUN4 but might be backed decisively by other putative interaction partners, such as the posterior localized LAP2 or lamin B1 (Göb et al., 2010; Schütz et al., 2005a). Vice versa, lamin B3 could also be critical for SUN4 localization, for example, as a recruiting platform, and thus organize its function in connecting the MT manchette to the NE, an issue that would be interesting to investigate in the future.

### SUN4 functions at the nucleocytoplasmic junction

Previous studies have shown that SUN4 is essential for sperm-head shaping and for correct assembly and NE attachment of the cytoplasmic MT manchette. Furthermore, upon SUN4 deficiency, its bona fide LINC complex partners SUN3 and nesprin-1 disappear from the NE, which demonstrates its crucial impact on the correct localization of other NE components (Calvi et al., 2015; Pasch et al., 2015). Here, we continued investigating this specific role of SUN4 by searching for further SUN4 *in vivo* interaction partners and studying the effect of a SUN4 depletion on other constituents of the posterior NE. Besides the lamins B1 and B3, which were extensively discussed above, SUN1 and LAP2 are two further NE components that are highly interesting in the context of nuclear shaping. Both, SUN1 and LAP2 (except for LAP2α, which is an isoform without a TM domain; see Vlcek et al., 1999) are integral proteins of the INM that, at least in the early steps of spermiogenesis, are localized at the posterior NE where they occupy territories that widely overlap with that of SUN4 (Alsheimer et al., 1998; Göb et al., 2010). This offers the opportunity for direct or indirect interaction with SUN4 or renders the possibility of a mutual interrelationship that might impact the territorial distribution of the other NE components.

Our co-IP results demonstrate that neither SUN1 nor LAP2 efficiently precipitates with SUN4 as bait, suggesting that they actually do not bind to SUN4. Nonetheless, we previously identified SUN4 as an essential determinant for SUN1 and nesprin-3 distribution (Pasch et al., 2015). In *Sun4*^−/−^ spermatids, we found the posterior SUN1 and nesprin-3 signals ‘leaking’ to the more lateral regions instead of being confined to the very posterior pole (Pasch et al., 2015; see also Fig. 8A,A′). Hence, SUN4 is apparently part of a kind of physical barrier for SUN1-containing LINC complexes, promoting their posterior confinement. This, however, applies overtly only for SUN1 and its partners. In the case of lamin B1, B3 and LAP2, no overt change in localization was observed in the *Sun4*^−/−^ background, demonstrating that their distribution is independent of the presence of SUN4.

Previous studies have disclosed that chromatin compaction in the spermatids is not substantially affected by SUN4 depletion (Calvi et al., 2015; Pasch et al., 2015). Given that the posterior pole of the spermatid nucleus is the only site where the chromatin tightly contacts the NE (Collas and Poccia, 1996), and LAP2 and lamin B1 are well-known to bind to chromatin, we suggest that the posterior confinement of LAP2, lamin B1 and lamin B3 might somehow be interrelated with the chromatin remodeling rather than being regulated by SUN4-containing LINC complexes. The INM protein DPY19L2, which localizes to the anterior pole of the spermatid nucleus, might be an essential coordinator here. As shown by Pierre et al. (2012), in DPY19L2-depleted spermatids, lamin B1 loses its posterior polarization and extends into the more apical regions. In addition, the acrosome and, remarkably, also the manchette fail to connect to the NE, indicating that proper SUN4 localization and general LINC complex distribution and function might depend on DPY19L2 and its partners. Investigating these interrelations would be another interesting challenge for future studies.

In conclusion, our present study provides clear evidence that SUN4 is an integral protein of the INM that has a typical SUN domain protein type II membrane topology and, therefore, in all probability, can not directly interact with any cytoskeletal proteins. SUN4 exclusively localizes to the lateral regions of the posterior spermatid NE, where it most likely forms heteromeric assemblies with SUN3. Via a specific interaction of its NTD with lamin B3, SUN4 anchors to the nuclear lamina and, together with SUN3, it forms the INM constituents of spermatid-specific LINC complexes with nesprin-1 as their putative KASH binding partner. Moreover, we also provide evidence that SUN4 is involved in regulating SUN3 expression. Together, our results contribute to a better understanding of the interactions and function of SUN4 at the spermatid nucleo-cytoplasmic junction and the entire process of sperm-head formation.

## MATERIALS AND METHODS

### Ethics statement

All animal care and experimental protocols were performed according to the guidelines specified within the German Animal Welfare Act (German Ministry of Agriculture, Health and Economic Cooperation). Animal housing and breeding were approved by the local regulatory agency of the city of Würzburg (reference ABD/OA/Tr; according to 111/1 No. 1 of the German Animal Welfare Act). All aspects of mouse work were carried out under strict guidelines to ensure careful, consistent and ethical handling of the mice.

### Animals and tissue preparation

Testis tissue samples were obtained either from wild-type, heterozygous or knockout litter mates of the *Sun4* knockout mouse (*Mus musculus*) strain (*Spag4^tm1(KOMP)Mbp^*) (Pasch et al., 2015) or from wild-type mice of other C57BL/6J strains. 8- to 16-week-old male mice were euthanized using CO_2,_ followed by cervical dislocation. Testes were dissected and further processed for protein analysis or immuno-localization studies as described below.

### Generation of plasmid constructs

To obtain Myc-S4_FL–EGFP and Myc-S4_ΔC–EGFP, the complete *Sun4* coding sequence (S4_FL) or the sequence coding for amino acids 1–195 (S4_ΔC) was amplified by PCR from the previously described full-length *Sun4* cDNA (Pasch et al., 2015) using the sequence-specific primers Sun4_inc.ATG_5′ and Sun4_SUNdom_3′_woStop or Sun4_delC-term_3′ (Table S2) and inserted into cloning vector pSC-B (Agilent Technologies, Waldbronn, Germany). After sequence verification, the respective fragments were excised with EcoRI and ligated into pCMV-Myc (Clontech Laboratories, Mountain View, CA). To generate the constructs coding for the double-tagged SUN4 peptide sequences, the resulting Myc-tagged versions were then cloned into the BamHI site of pEGFP-N3 (Clontech Laboratories). Finally, to eliminate an undesired TAG codon that was introduced with the cloning procedure between the 5′ Myc tag sequence and the SUN4 coding region, we performed site-directed mutagenesis-PCR to replace the TAG with CAG (primers: Muta_Myc-S4_5′ and Muta_Myc-S4_3′; Table S2).

Myc–S4_ΔHM1_ΔC–EGFP, Myc–S4_ΔHM2_ΔC–EGFP, Myc–S4_ΔHM1_ΔHM2_ΔC–EGFP were generated from the S4_ΔC-pCMV-Myc construct by deletion PCR using primers Sun4_delTM1_3′, Sun4_TM2_5′ (for deletion of HM1) or Sun4_cc_5′, Sun4_delTM2_3′ (for deletion of HM2) (Table S2).

For generating S4_FL–EGFP and S4_ΔC–EGFP, the respective *Sun4* fragments were cut out from Myc-S4-pEGFP-N3 or Myc-S4_ΔC-pEGFP-N3 and cloned into the EcoRI site of pEGFP-N2. EGFP-tagged HM deletion constructs were generated from S4_ΔC-EGFP as described above.

To generate S1_FL–EGFP, the *Sun1* coding sequence was amplified from *Sun1* full-length cDNA (Göb et al., 2010) with primers Sun1_5′ and Sun1_3′_woStop (Table S2) and cloned into the SmaI site of pEGFP-N2.

### Expression analysis by RT-PCR

To analyze *Sun3*, *Sun4* and, as a control, *Gapdh* transcripts in the *Sun4^+/+^*, *Sun4^+/−^* and *Sun4^−/−^* mouse testes, total RNA was extracted from testicular cell suspensions of adult (12 weeks old) mice of the respective genotypes using peqGOLD TriFast reagent (PEQLAB, Erlangen, Germany) according to the manufacturer's protocol. Reverse transcription was performed on 1.5 µg of total RNA with oligo(dT) primer (Thermo Fisher Scientific, Life Technologies, Darmstadt, Germany) and M-MLV reverse transcriptase (Promega, Mannheim, Germany). cDNAs were amplified from 1 µl reverse transcriptase reactions each according to standard protocols. *Sun3* and *Sun4* cDNAs were amplified in 25 cycles using primers Sun3_RT_5′ and Sun3_RT_3′ (annealing temperature, 55°C) or Sun4_cc_5′ and Sun4_cc_3′ (annealing temperature, 54°C), respectively. *Gapdh* cDNA was amplified in 29 cycles with primers GAPDH5′ and GAPDH3′ (annealing temperature, 55°C). Primer sequences are listed in Table S2. Quantification of relative mRNA levels was performed using ImageJ (http://imagej.nih.gov/ij), version 1.53n. For each gene, equally sized areas covering the reverse transcriptase signals were defined with the ROI Manager and signal intensities were measured using the ROI measure function. Relative *Sun3* levels were normalized to the respective *Gapdh* expression levels. The test for statistical significance was undertaken with a two-tailed one-sample *t*-test using GraphPad Quickcalcs (https://www.graphpad.com/quickcalcs/oneSampleT1/).

### Cell culture and transfection

For our experiments, we used COS-7 (DSMZ; ACC-60) and NIH 3T3 (ATCC; CRL-1658) cells. Cells were grown in Dulbecco's modified Eagle's medium (DMEM; Gibco by Life Technologies, Darmstadt, Germany) supplemented with 10% fetal calf serum (FCS; Capricorn Scientific, Ebsdorfergrund, Germany) and 1% penicillin-streptomycin (Thermo Fisher Scientific, Dreireich, Germany) at 37°C and 5% CO_2_. Cell lines were regularly tested for contamination with mycoplasma.

COS-7 cells were transfected with the respective Myc-Sun4-pEGFP fusion constructs using Effectene™ (Qiagen, Hilden, Germany) following the manufacturer's protocol and incubated overnight before analysis of the ectopically expressed fusion proteins.

Transfection of NIH 3T3 cells was carried out using the Matra™ magnetic bead transfection system (IBA, Göttingen, Germany) to warrant nuclear membrane integrity for subsequent FRAP experiments. To enhance transfection efficiency, NIH 3T3 cells were treated twice in a row with the respective construct in the prepared transfection mixture.

Before analysis, cells were further incubated for ∼48 h at 37°C and 5% CO_2_.

### Antibodies

Primary antibodies used in this study were: guinea pig and rabbit anti-SUN4, guinea pig anti-SUN3, guinea pig anti-SUN1, rabbit anti-lamin B1, and rabbit anti-lamin B3, mouse anti-LAP2, rabbit anti-laminA/C, mouse anti-actin, mouse anti-PDI RL90, mouse anti-GFP (B-2) and mouse anti-Myc.

Secondary antibodies were: Alexa Fluor 488 goat anti-guinea pig-IgG, Alexa Fluor 488 goat anti-rabbit-IgG, Texas red goat anti-mouse, horseradish peroxidase goat anti-guinea pig-IgG, -rabbit-IgG or -mouse-IgG, and 6 nm gold donkey anti-guinea pig-IgG and goat anti-rabbit-IgG.

Detailed information about sources and working dilutions of the antibodies used in this study is listed in Tables S3 and S4.

### Immunocytochemistry

#### Immunofluorescence labeling of tissue sections

Halved testes were fixed in 1% PBS-buffered formaldehyde (FA) for 3 h at room temperature and further processed for paraffin embedding as described in Alsheimer et al. (2005). Embedded tissues were cut into 3–6 µm sections using a paraffin microtome (Leitz, Wetzlar, Germany) and placed on SuperFrost^®^ Plus slides (Menzel Gläser, Thermo Fisher Scientific, Darmstadt, Germany). Deparaffination and antigen retrieval was performed as described previously (Link et al., 2016). Specimens were permeabilized with 0.1% Triton X-100 in PBS for 10 min, and washed in PBS and blocked with PBT (0.15% BSA, 0.1% Tween 20 in PBS) for 1 h. The samples were incubated with the appropriate primary antibodies (dissolved in PBT) for 1 h at room temperature or, depending on the antibody, overnight at 4°C, washed with PBS and then incubated with corresponding fluorescently labeled secondary antibodies (dissolved in PBS) for 30 min. DNA was counterstained with Hoechst 33258 (Serva, Heidelberg, Germany).

#### Immunofluorescence labeling of transfected culture cells

Transfected cells were fixed with 1% FA in PBS for 3 min, permeabilized with 0.1% Triton X-100 in PBS for 5 min and blocked for 90 min with PBT at room temperature. Incubation with primary and secondary antibodies was performed as described above.

#### Immunogold labeling

Immunogold labeling was performed either on sections of shock frozen native testis-tissue or on sections of pre-fixed TissueTek^®^ O.C.T Compound embedded (Sakura, Staufen, Germany) testes. For embedding in TissueTek^®^ O.C.T Compound, dissected testes were cut into halves and fixed for 1 h at room temperature in 1% PBS buffered FA, followed by two washing steps in PBS for 30 min each. The fixed tissue was then dehydrated by consecutive soaking in 15% and 30% sucrose solution (in PBS), embedded in TissueTek^®^ O.C.T Compound and frozen in precooled (−140°C) methyl butane. The frozen tissue was sectioned to 7–10 µm using a 2800 Frigocut E cryo-microtome (Reichert-Jung; Leica Instruments, Nussloch, Germany). Sections of the native tissue were fixed in 1% FA (in PBS). Fixed tissue of both types was permeabilized with 0.05% Triton X-100 (in PBS) for 8–10 min, washed in PBS and blocked with PBT for 60–90 min at room temperature. Incubation with primary antibodies was performed with anti-SUN4 rabbit antibody overnight at 4°C or with anti-SUN4 guinea pig antibody for 1 h at room temperature. Following washing in PBS, samples were subjected to corresponding gold-conjugated secondary antibodies for 2 h at room temperature (anti-rabbit-IgG) or overnight at 4°C (anti-guinea pig-IgG).

### *In situ* proteinase K digestions

*In situ* proteinase K (Serva, Heidelberg, Germany) digestions were performed according to Liu et al. (2007). Cos7 cells were transfected with Myc- and EGFP double-tagged Sun4 constructs (four 35-mm dishes for each construct). At 24 h after transfection, the cells were washed twice with ice-cold KHM buffer (110 mM KOAc, 2 mM MgCl_2_, 20 mM HEPES, pH 7.4) for 1 min each. One well per construct was incubated with 4 µg/ml proteinase K in KHM, one with 4 µg/ml proteinase K and 0.5% Triton X-100 in KHM and a third one in KHM without any additives, each for 45 min at room temperature. A fourth well of each construct was first incubated with 24 µM ice-cold digitonin in KHM for 10 min, washed in KHM, and subsequently digested in 4 µg/ml proteinase K solution for 45 min. After digestion, 40 µg/ml PMSF and 1:100 protease inhibitor mix (stock solution: 5 mM α-aminocaproic acid, 1 mM benzamidine, 1 mM EDTA, 2 μg/ml aprotinin, 2 μg/ml antipain, 2 μg/ml chymostatin, 2 μg/ml leupeptin and 2 μg/ml pepstatin) were added. Cells were transferred into reaction tubes, washed in KHM, and boiled in 2× SDS-sample buffer (120 mM Tris-HCl pH 6.8, 10% SDS, 20% glycerol, 20% 2-mercaptoethanol and Bromophenol Blue) at 95°C for 5–10 min. The samples were separated by SDS-PAGE, transferred to nitrocellulose membranes and probed with anti-Myc and anti-GFP antibodies.

### Co-immunoprecipitation

Co-IP experiments were performed with testicular suspension cells of 9- to 16-week-old mice. For co-IPs of lamin B3 with SUN4, we lysed the cells in RIPA buffer (1% Triton X-100, 0.5% sodium deoxycholate, 0.1% SDS, 1 mM β-glycerophosphate, 1 mM Na_3_VO_4_, 1 mM EDTA and 1 mM EGTA, in PBS) supplemented with 1× proteinase inhibitor cocktail (#04693132001; Roche Diagnostics, Mannheim, Germany) for 20 min on ice with occasional inverting. The lysate was cleared by centrifugation at 14,000 ***g*** and 4°C. The supernatant was divided into two equal aliquots, and each aliquot was incubated with either guinea pig anti-SUN4 antibody or non-specific guinea pig-IgG (1 µg antibody per ∼10^8^ cell equivalents each), before being incubated on a rocker overnight. Immuno-complexes were pulled with Dynabeads™ Protein G (10003D, Thermo Fisher Scientific, Life Technologies, Darmstadt, Germany); 1.0 mg RIPA-pre-equilibrated beads per ∼10^8^ cell equivalents were added to each aliquot, and the lysate–bead mixtures were incubated on a rocker for 2 h at 4°C.

The protein-loaded beads were harvested using a magnet and washed three times with 250 µl RIPA buffer. The supernatant was used as a control for pulldown efficiency. Proteins contained in the supernatant were precipitated with acetone according to standard procedures. Precipitated proteins and immunocomplex-decorated beads were resuspended in 2× SDS-sample buffer and heated to 95°C for 15 min. Denatured samples were analyzed by SDS-PAGE and western blotting.

For detection of interactions of SUN4 with other transmembrane proteins, we implemented the following adaption. Prior to co-IP, cellular membranes and membrane-associated proteins were extracted from ∼2×10^8^ cells by lysis in 5 ml urea buffer (8 M urea, 100 mM Tris-HCl pH 8.0 and 1 mM DTT) for 20 min at room temperature. The lysate was centrifuged at 100,000 ***g*** and 4°C for 1 h to precipitate the membranes. The resulting pellet, including the integral membrane proteins, was repeatedly washed with PBS supplemented with protease inhibitor cocktail and reconcentrated using an Amicon^®^ Ultra-0.5 device with 10 K MWCO (Merck) until residual urea was diluted below 10 mM. Then the pellet was steeped in 2 ml RIPA buffer at 4°C overnight with continuous rocking to extract protein complexes from the membranes. Insoluble pellet fragments were removed by centrifugation at 15,000 ***g*** and 4°C. Dissolved immunocomplexes were collected as described above, using 2 µg of rabbit anti-SUN4 antibody and unspecific rabbit IgG, respectively, per ∼10^8^ cell equivalents.

### SDS-PAGE and western blotting

Protein samples were resuspended in 2× SDS-sample buffer, denatured at 95°C for several minutes and separated by SDS-PAGE according to standard procedures. For western blotting, proteins were transferred to nitrocellulose membranes. Membranes were blocked in TBST buffer (10 mM Tris-HCl pH 7.4, 150 mM NaCl, 0.1% Tween 20) supplemented with 10% dry milk powder overnight at 4°C and incubated for 60–90 min at room temperature with respective primary antibodies in blocking solution. After three 10 min washes in TBST buffer, the membranes were incubated with corresponding peroxidase-coupled secondary antibodies. Bound antibodies were detected with chemiluminescent substrate (Western Lightning^®^ Plus, Perkin Elmer, Waltham, MA) and visualized with iBright™ CL1000 (Thermo Fisher Scientific, Life Technologies, Darmstadt, Germany). Figs 6, 7 and Fig. S3 show cropped blots. Full, uncropped pictures of the respective blotting membranes are shown in Fig. S4 (blot transparency). Relative protein expression levels were quantified with ImageJ version 1.53n as was described above for mRNA levels. SUN3 protein levels were normalized to the relative amount of actin in the same probes. Test for statistical significance was performed with two-tailed one-sample *t*-tests using GraphPad Quickcalcs (https://www.graphpad.com/quickcalcs/oneSampleT1/).

### Fluorescence microscopy and FRAP

Fluorescence images were acquired on a Leica TCS-SP2 AOBS confocal laser scanning microscope with a 63×/1.40 HCX PL APO oil-immersion objective and a pinhole setting of 1.0 P AU (Leica Microsystems, Wetzlar, Germany), using an argon/krypton (Ar/Kr) laser for excitation at 488 nm and two diode lasers for excitation at wavelengths 405 nm and 561 nm. The respective fluorescence emissions were detected at 495–565 nm, 413–569 nm and 592–690 nm. Images shown are maximum projections of three sequential images. Images were processed with ImageJ (http://imagej.nih.gov/ij) and Adobe Photoshop CS5 (Adobe Systems, San Jose, CA).

For FRAP experiments, the transiently transfected cells were analyzed in FluoroDish culture dishes (World Precision Instruments, Sarasota, FL) using the 488 nm laser line of the Ar/Kr laser with pinhole setting 1.6 P AU. Fluorescence emission of the EGFP protein was detected at 494–563 nm. FRAP measurements were performed according to Jahn et al. (2010). Two single pre-bleach scans were acquired, followed by eight bleach pulses (100% laser intensity) within a circular bleach spot of 2 µm diameter. Recovery data were collected from single section images with 2× accumulation at 1 s (30 images), 2 s (30 images) and 5 s intervals (35 images). For imaging, the laser power was set to 7% of bleach intensity. Resulting intensity values of fluorescence emission were background-subtracted according to Phair and Misteli (2000), using LCS Leica Confocal Software (Copyright 1997-2004 by Leica Microsystems). Corresponding FRAP curves were generated in GraphPad Prism version 9.0.1 (and following) for Windows (GraphPad Software, San Diego, CA, USA). Presented recovery values are means±s.d. of 10–15 different cells pooled from at least five independent transfection experiments. Only cells of sufficient quality and comparable fluorescence intensity were considered in the statistics. Time points at 50% signal recovery (t_50_ values) were calculated as follows:
(1)

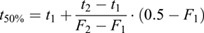
where *t*_50%_ is the time of 50% fluorescence recovery, *t*_1_ the last timepoint before 50% recovery, *t*_2_ the first timepoint after 50% recovery, *F*_1_ the fluorescence intensity at *t*_1_, and *F*_2_ the fluorescence intensity at *t*_2_.

Statistical analysis, including tests for statistical significance, was undertaken with one-way ANOVA followed by Tukey's multiple comparisons test using GraphPad Prism version 9.0.1 (and following). Corresponding box-and-whisker plots were also generated with GraphPad Prism.

### Electron microscopy

Immunogold-labeled testes sections were fixed in 2.5% glutaraldehyde solution (2.5% glutaraldehyde, 50 mM KCl, 2.5 mM MgCl, 50 mM cacodylate; pH 7.2) for 45 min, subsequently washed in 50 mM cacodylate buffer (pH 7.2) and post-fixed with 1–2% osmium tetroxide in 50 mM cacodylate for 1 h on ice. Following several washing steps in H_2_O, the samples were dehydrated on ice in an increasing ethanol series, incubated two times in propylene oxide for 5 min each, and embedded in Epon as described in Link et al. (2016). Ultrathin tissue sections (50–60 nm) were cut on a Leica EM UC7 ultramicrotome and transferred onto copper grids (50 mesh). Sections were stained with uranyl acetate and lead citrate according to standard procedures. Samples were analyzed with a JEOL JEM-2100 transmission electron microscope operated at 200 kV (Jeol, Eching, Germany).

## Supplementary Material

10.1242/joces.260155_sup1 Supplementary information
