# Supplementary material for: SUN4 is a spermatid type II inner nuclear membrane protein that forms heteromeric assemblies with SUN3 and interacts with lamin B3
Source: J Cell Sci. 2023 Mar 27;136(7):jcs260155. doi: 10.1242/jcs.260155 (PMC10112980; doi:10.1242/jcs.260155)
Supplement: Supplementary information [file joces-136-260155-s1.pdf]

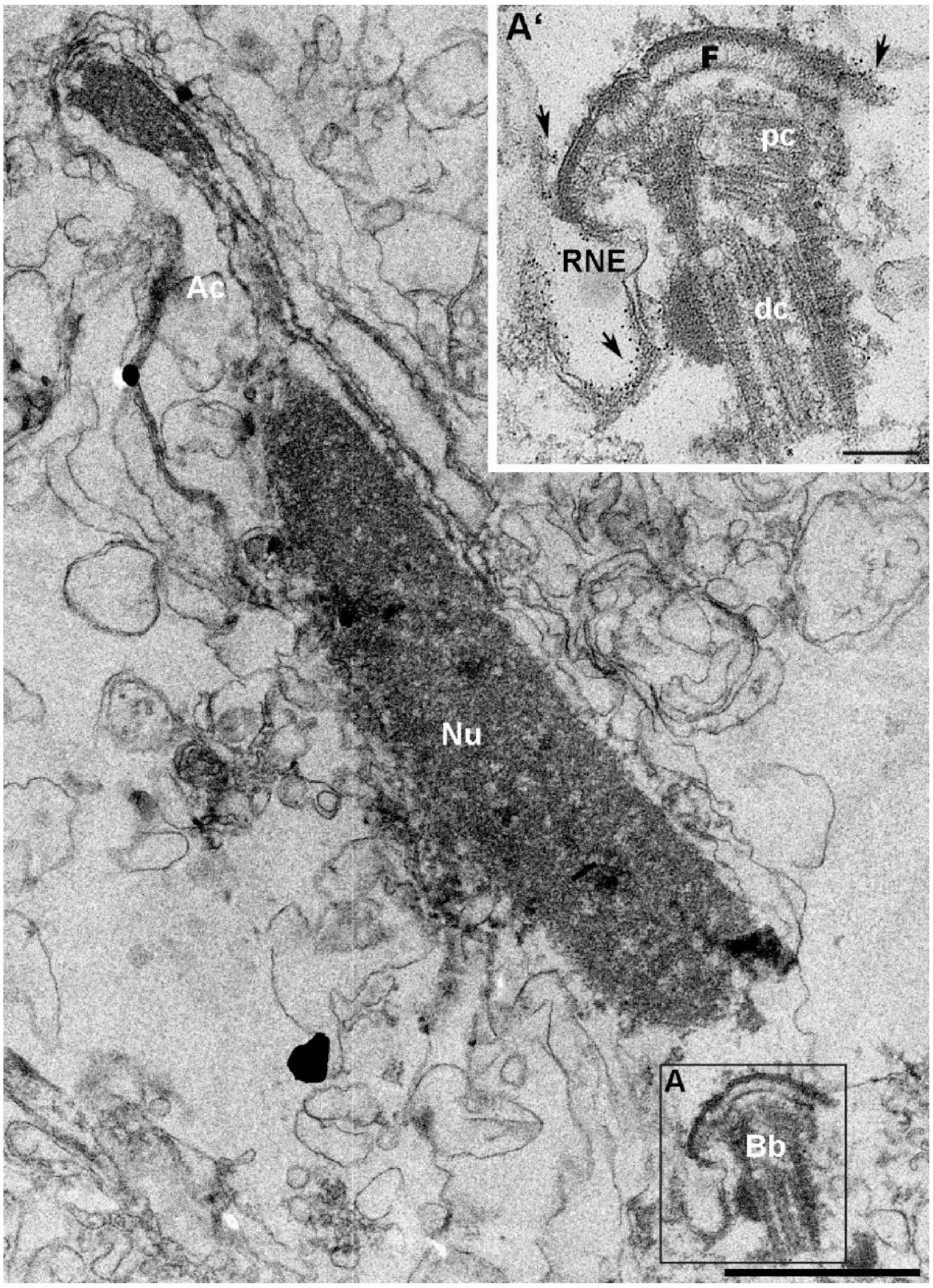


**Fig. S1. The SUN4 N-terminal domain is localized at the RNE of elongating spermatids.** Representative electron micrographs of elongating spermatids in native mouse testis cryo-sections with immunogold-labelling (6 nm colloidal gold) of the SUN4 NTD. Area A is enlarged in A'. Gold particles (arrows) are mainly localized along the inner membrane of the redundant nuclear envelope and to both sides of the fossa region, but not directly within the implantation fossa (A'). Scale bars: 1 µm (A) and 200 nm (A'). Bb, basal body; Nu, nucleoplasm; Ac, acrosome; F, implantation fossa; RNE, redundant nuclear envelope, dc, distal centriole; pc, proximal centriole. Image is re-presentative of two experimental repeats.

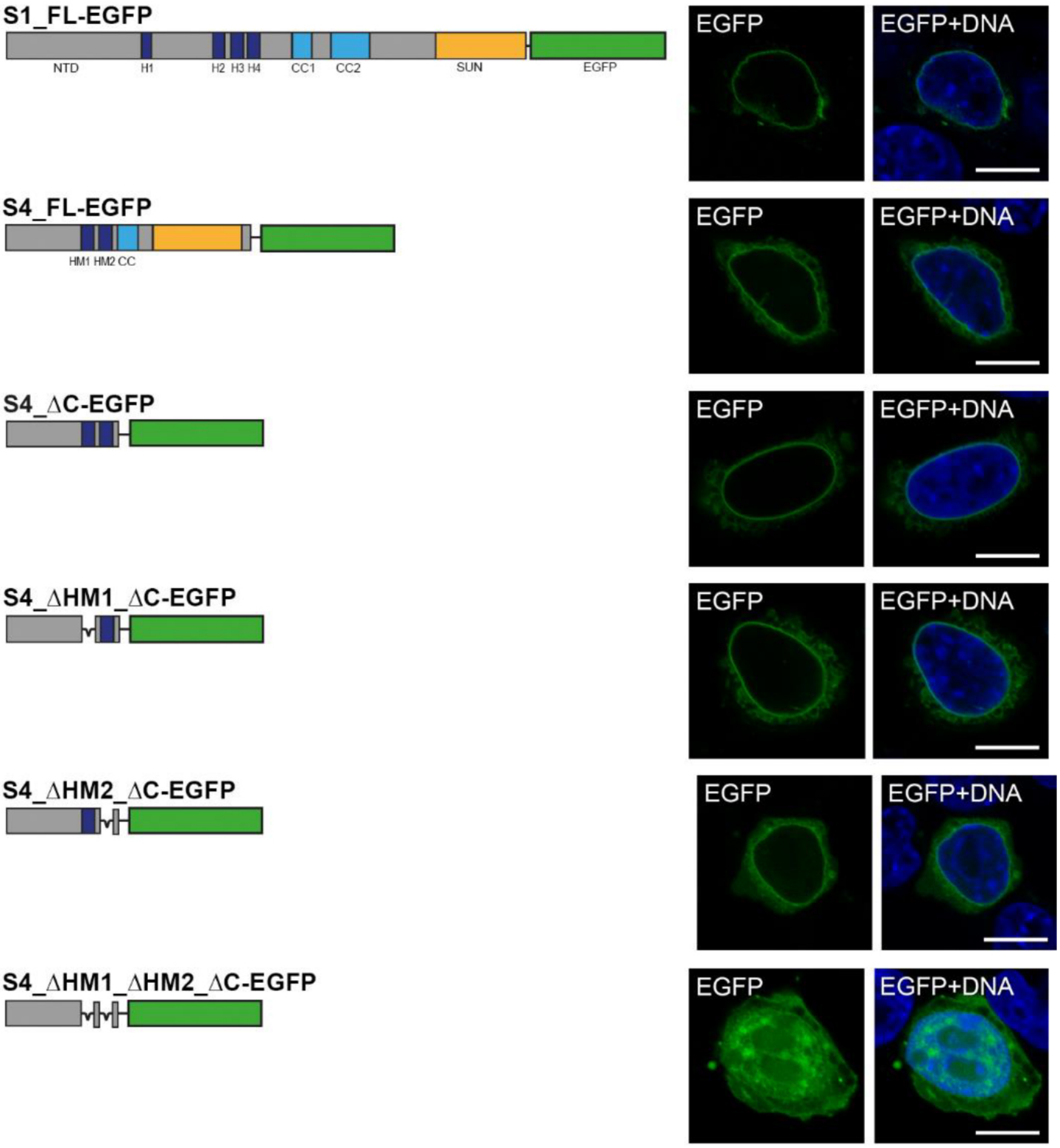


**Fig. S2. Subcellular localization of EGFP-tagged SUN1 and SUN4 reporter constructs used for FRAP analysis.** EGFP-tagged full-length SUN1 and SUN4, as well as SUN4 constructs with deletion of the C-terminal coiled-coil and SUN domain were transiently transfected into NIH 3T3 cells. Confocal microscopy was used to monitor their expression patterns 48 h after transfection via their EGFP-tag. For each construct, a fluorescence image of one representative cell is shown. DNA was counterstained with Hoechst. Scale bars: 10 µm. Images are reprensentative for at least five independent transfection experiments.

Journal of Cell Science • Supplementary information

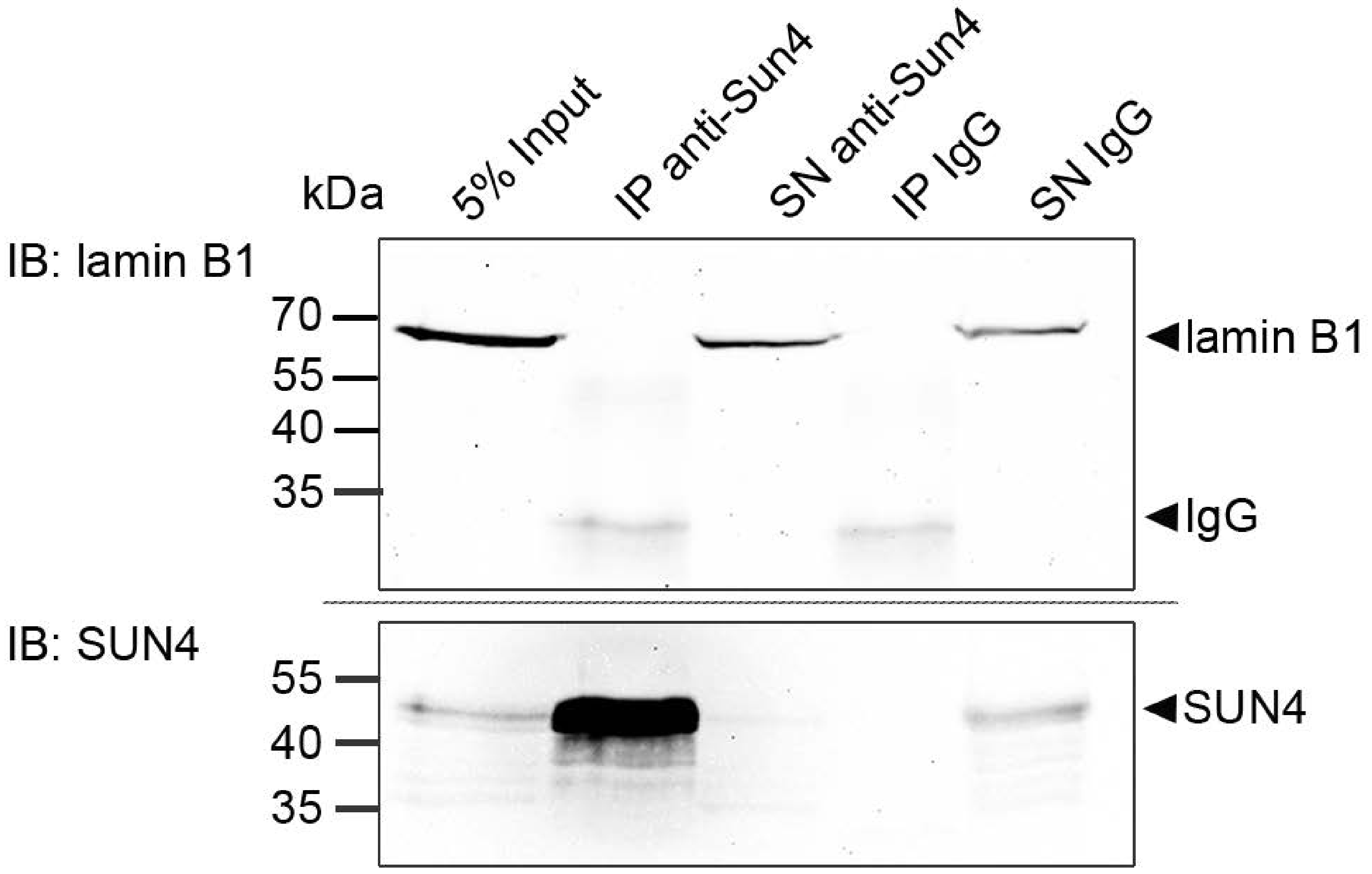


**Fig. S3. SUN4 does not appear to bind to lamin B1 *in vivo.*** Mouse testicular suspension cells were lysed in RIPA buffer and subjected to co-immunoprecipitation with guinea pig anti-SUN4 NTD antibody and unspecific guinea pig IgG as a negative control. The precipitates (IP) and supernatants containing unbound proteins (SN) were analyzed by immunoblotting (IB) using a rabbit anti-lamin B1 antibody. The SUN4 control presented here is identical to that in Fig. 7, as SUN4-lamin B1 interaction was analysed on the same co-IP samples used to test for SUN4-lamin B3 interaction. It is just shown here again for better visual comparison. Untreated testis cells served as input control. Amounts of loaded cell equivalents: 5% Input=$2x10^6$; IP=$4x10^7$; SN=$5x10^6$. Blot shown is representative for three experimental repeats.

Journal of Cell Science • Supplementary information

## Figure 6A

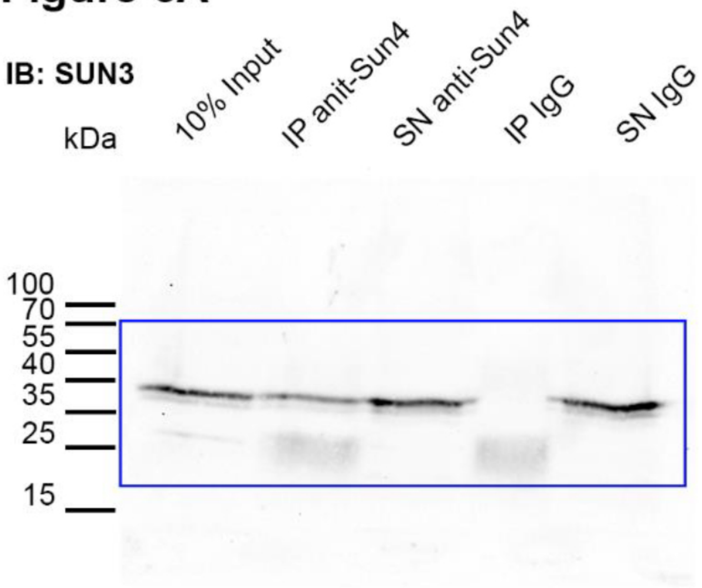


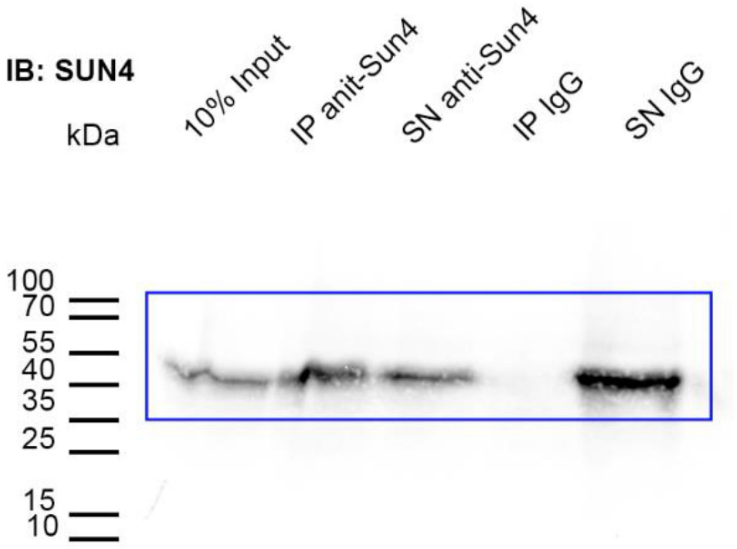


## Figure 6B

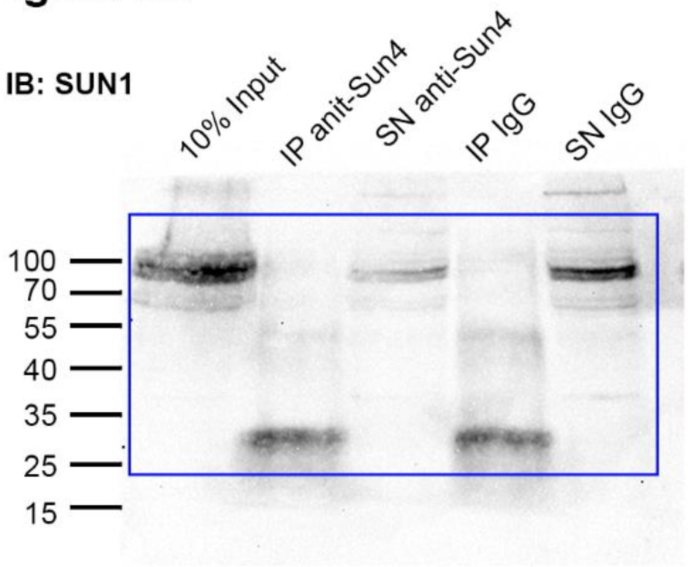


## Figure 6C

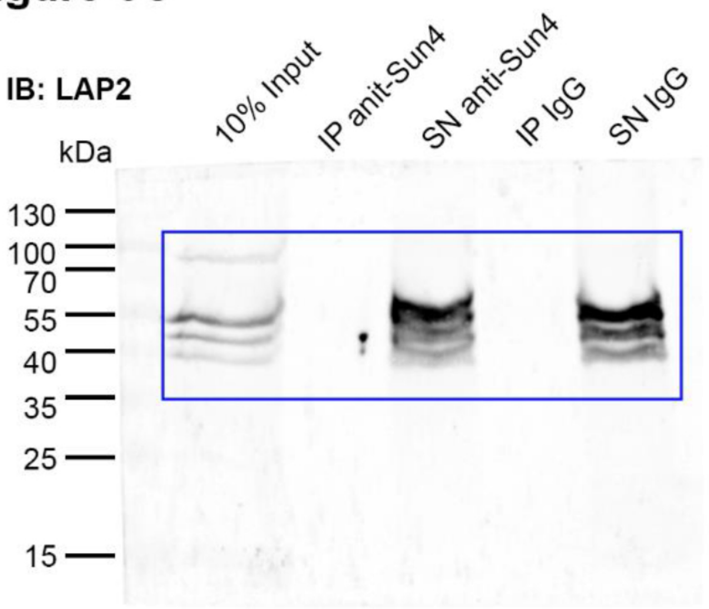


## Figure 6D

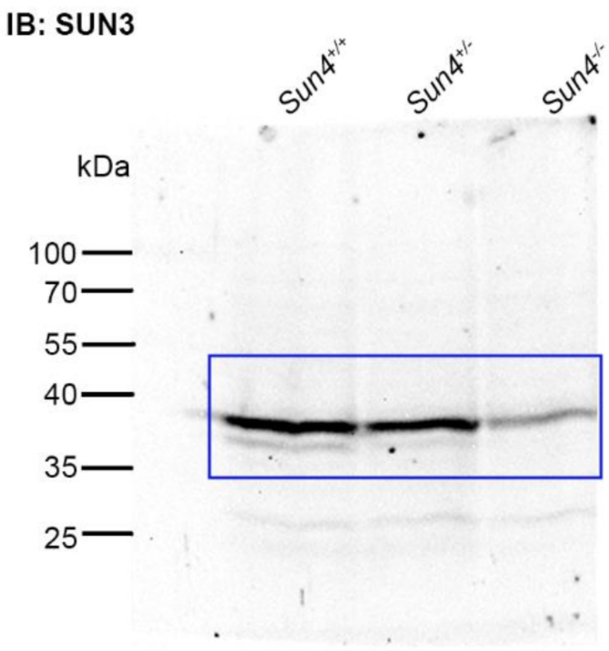


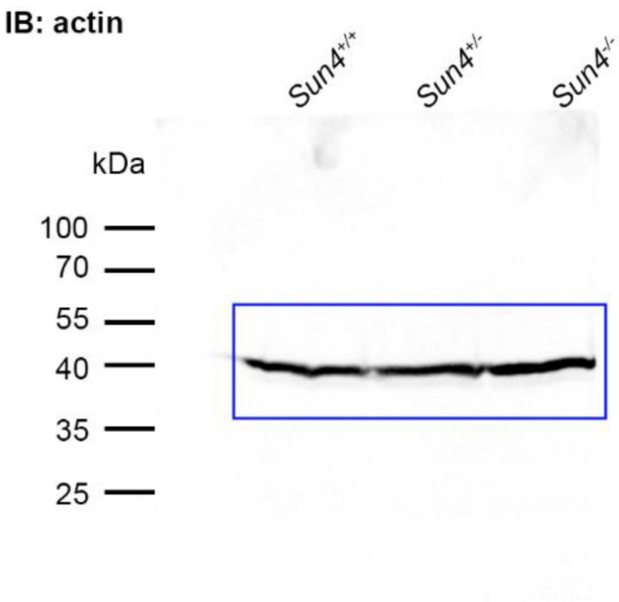


**Fig. S4. Blot transparency** (continued on the next page)

Journal of Cell Science • Supplementary information

## Figure 7

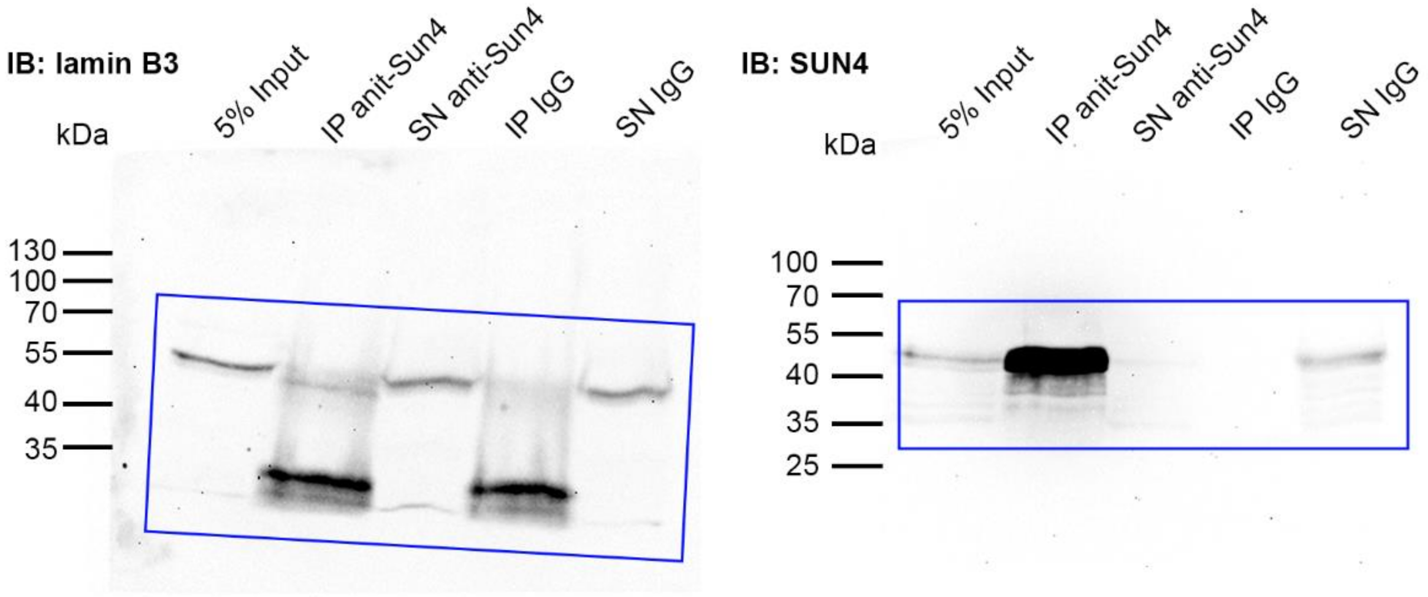


## Figure S3

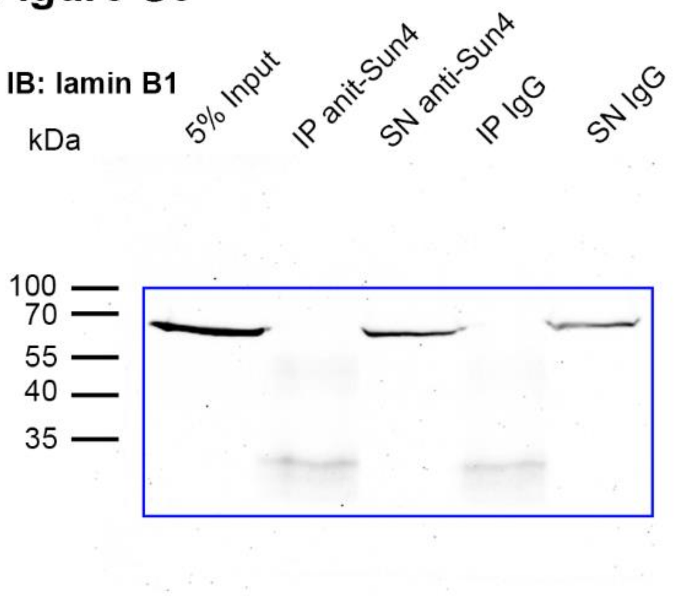


**Fig. S4. Blot transparency.** Full, uncropped images of all cropped (blue rectangles) Western Blots presented in this study. Reference figure numbers are indicated above the images.

Journal of Cell Science • Supplementary information

# Table S1. Details on statistical analysis of FRAP experiments (Output of GraphPad Prism multiple comparisons analysis)

## Ordinary one-way Anova of t50%-values - Multiple Comparisons

| | |
|---|---|
| Number of families | 1 |
| Number of comparisons per family | 3 |
| Alpha | 0.05 |

| **Tukey's multiple comparisons test** | Mean Diff. | 95.00% CI of diff. | Below threshold? | Summary | Adjusted P Value |
|---|---|---|---|---|---|
| S4_ΔC-EGFP vs. S4_ΔHM1_ ΔC-EGFP | 2.745 | 0.8906 to 4.99 | Yes | ** | 0.0024 |
| S4_ΔC-EGFP vs. Sun4_ΔHM2_ ΔC-EGFP | 4.903 | 3.049 to 6.757 | Yes | **** | <0,0001 |
| S4_ΔHM1_ ΔC-EGFP vs. Sun4_ΔHM2_ ΔC-EGFP | 2.158 | 0.3042 to 4.013 | Yes | * | 0.0192 |

| **Test details** | Mean 1 | Mean 2 | Mean Diff. | SE of diff. | n1 | n2 | q | DF |
|---|---|---|---|---|---|---|---|---|
| S4_ΔC-EGFP vs. S4_ΔC_ ΔHM1-EGFP | 12.03 | 9.289 | 2.745 | 0.7632 | 15 | 15 | 5.086 | 42 |
| S4_ΔC-EGFP vs. Sun4_ΔC_ ΔHM2-EGFP | 12.03 | 7.131 | 4.903 | 0.7632 | 15 | 15 | 9.086 | 42 |
| S4_ΔC_ ΔHM1-EGFP vs. Sun4_ΔC_ ΔHM2-EGFP | 9.289 | 7.131 | 2.158 | 0.7632 | 15 | 15 | 4 | 42 |

## Ordinary one-way Anova of fluorescence intensities 30 s post-bleaching - Multiple Comparisons

| | |
|---|---|
| Number of families | 1 |
| Number of comparisons per family | 6 |
| Alpha | 0.05 |

| **Tukey's multiple comparisons test** | Mean Diff. | 95.00% CI of diff. | Below threshold? | Summary | Adjusted P Value |
|---|---|---|---|---|---|
| S4_ΔC-EGFP vs. S4_ΔHM1_ΔC-EGFP | -0.0502 | -0.09558 to -0.004812 | Yes | * | 0.0247 |
| S4_ΔC-EGFP vs. S4_ΔHM2_ΔC-EGFP | -0.05253 | -0.09791 to -0.007142 | Yes | * | 0.0172 |
| S4_ΔC-EGFP vs. S4_ΔHM1_ΔHM2_ΔC-EGFP | -0.2849 | -0.3330 to -0.2367 | Yes | **** | <0,0001 |
| S4_ΔHM1_ΔC-EGFP vs. S4_ΔHM2_ΔC-EGFP | -0.00233 | -0.04771 to 0.04305 | No | ns | 0.9991 |
| S4_ΔHM1_ΔC-EGFP vs. S4_ΔHM1_ΔHM2_ΔC-EGFP | -0.2347 | -0.2828 to -0.1865 | Yes | **** | <0,0001 |
| S4_ΔHM2_ΔC-EGFP vs. S4_ΔHM1_ΔHM2_ΔC-EGFP | -0.2323 | -0.2805 to -0.1842 | Yes | **** | <0,0001 |

| **Test details** | Mean 1 | Mean 2 | Mean Diff. | SE of diff. | n1 | n2 | q | DF |
|---|---|---|---|---|---|---|---|---|
| S4_ΔC-EGFP vs. S4_ΔHM1_ΔC-EGFP | 0.686 | 0.7362 | -0.0502 | 0.01711 | 15 | 15 | 4.149 | 53 |
| S4_ΔC-EGFP vs. S4_ΔHM2_ΔC-EGFP | 0.686 | 0.7385 | -0.05253 | 0.01711 | 15 | 15 | 4.341 | 53 |
| S4_ΔC-EGFP vs. S4_ΔHM1_ΔHM2_ΔC-EGFP | 0.686 | 0.9708 | -0.2849 | 0.01815 | 15 | 12 | 22.2 | 53 |
| S4_ΔHM1_ΔC-EGFP vs. S4_ΔHM2_ΔC-EGFP | 0.7362 | 0.7385 | -0.00233 | 0.01711 | 15 | 15 | 0.1926 | 53 |
| S4_ΔHM1_ΔC-EGFP vs. S4_ΔHM1_ΔHM2_ΔC-EGFP | 0.7362 | 0.9708 | -0.2347 | 0.01815 | 15 | 12 | 18.29 | 53 |
| S4_ΔHM2_ΔC-EGFP vs. S4_ΔHM1_ΔHM2_ΔC-EGFP | 0.7385 | 0.9708 | -0.2323 | 0.01815 | 15 | 12 | 18.1 | 53 |

Journal of Cell Science • Supplementary information

**Table S2. Oligonucleotides used in this study.**

| Primer | Sequence | Purpose |
|---|---|---|
| **Sun4_inc.ATG_5’** | 5‘-AGGTCAGGATGCGGCGGA-3‘ | 5’ primer for amplification of *Sun4* and S4_ΔC cDNAs to create entry StrataClone construct |
| **Sun4_SUNdom_3’_woStop** | 5‘-ATGGGGTCCCCCTGTGA-3‘ | 3’ primer for amplification of S4_FL cDNA to create StrataClone construct |
| **Sun4_delC-term_3’** | 5‘-CAGCATCTCCGTAGGTTCGTT-3‘ | 3’ primer for amplification of S4_ΔC cDNA to create StrataClone construct |
| **Sun4_delTM1_3’** | 5‘-CCTCGGGGTAGGCATCTC-3‘ | 3’ primer for deletion of HM1 |
| **Sun4_TM2_5'** | 5‘-TGCAGGGAAATCTGCTCC-3‘ | 5’ primer for deletion of HM1 |
| **Sun4_delTM2_3'** | 5‘-GGAGCAGATTTCCCTGCA-3‘ | 3’ primer for deletion of HM2 |
| **Sun4_cc_5'** | 5‘-CCTTTGGAGAACGAACCTACG -3‘ | 5’ primer for deletion of HM2;<br>5‘ primer for *Sun4* RT-PCR expression analysis |
| **Muta_Myc-S4_5’** | 5’-CCGAATTCGCCCTCAGGTCAGGATGC-3’ | 5’ primer for site-directed mutagenesis to eliminate undesired TAG-codon |
| **Muta_Myc-S4_3’** | 5’- GCATCCTGACCTGAGGGCGAATTCGG-3‘ | 3’ primer for site-directed mutagenesis to eliminate TAG-codon |
| **Sun1_5’** | 5’-ATGGACTTTTCTCGGCTGCAC-3’ | 5’ primer for amplification of *Sun1* coding sequence |
| **Sun1_3’_woStop** | 5’-CTGGATGGGCTCTCCGTG-3’ | 3’ primer for amplification of *Sun1* coding sequence |
| **Sun3_RT_5’** | 5‘-ACAGAGTTTCCTCAAAAACCCAG-3‘ | 5‘ primer for *Sun3* RT-PCR expression analysis |
| **Sun3_RT_3’** | 5‘-AGCCAACTGTATTTGGTCCCCT-3‘ | 3‘ primer for *Sun3* RT-PCR expression analysis |
| **Sun4_cc_3‘** | 5‘-GTCAGGTTTGCGCACGAAGT-3‘ | 3‘ primer for *Sun4* RT-PCR expression analysis |
| **GAPDH5‘** | 5‘-GGGCCCACTTGAAGGGTGGAGC-3‘ | 5‘ primer for *Gapdh* RT-PCR expression analysis |
| **GAPDH3‘** | 5‘-GTCAGATCCACGACGGACACATTGG-3‘ | 3‘ primer for *Gapdh* RT-PCR expression analysis |

Journal of Cell Science • Supplementary information

**Table S3. Primary antibodies used in this study.** IF, immunofluorescence; EM, electron microscopy (immunogold labeling); IB, immunoblot; mAb, monoclonal antibody; pAb, polyclonal antibody.

| Antibody | Manufacturer (Catalogue number/ Reference) | Host | Working dilution | | |
|---|---|---|---|---|---|
| | | | IF | EM | IB |
| **actin** | Sigma-Aldrich (A4700) | Mouse mAb | | | 1:500 |
| **GFP (B-2)** | Santa Cruz Biotechnologies (sc-9996) | Mouse mAb | 1:200 | | 1:200 |
| **lamin A/C** | Santa Cruz (sc-20681) | Rabbit pAb | | | 1:2,000 |
| **lamin B1** | Sigma-Aldrich (ZRB1143) | Rabbit mAb | 1:100 | | 1:5,000 |
| **lamin B3** | Own synthesis (Schütz et al., 2005) | Rabbit pAb | 1:100 | | |
| **13d4 (LAP2)** | Own synthesis (Alsheimer et al., 1998) | Mouse mAb | Undiluted hybridoma cell supernatant | | |
| **Myc** | Thermo Fisher Scientific (9E10) | Mouse mAb | 1:200 | | 1:8,000 |
| **PDI RL90** | Thermo Fisher Scientific (MA3-019) | Mouse mAb | 1:200 | | 1:1,000 |
| **SUN1** | Own synthesis (Göb et al., 2010) | Guinea pig pAb | 1:800 | | 1:5,000 |
| **SUN3** | Own synthesis (Göb et al., 2010) | Guinea pig pAb | 1:1000 | | 1:5,000 |
| **SUN4 peptide** | Own synthesis (Pasch et al., 2015) | Rabbit pAb | | | 1:5,000 |
| **SUN4-NTD** | Own synthesis (Pasch et al.,2015) | Guinea pig pAb | 1:2000 | 1:400 | 1:5,000 |
| **SUN4-NTD** | Own synthesis (Pasch et al.,2015) | Rabbit pAb | | 1:2400 | |
| **IgG** | Jackson ImmunoResearch Laboratories (011-000-003) | Rabbit pAb | | | |
| **IgG** | Jackson ImmunoResearch Laboratories (006-000-003) | Guinea pig pAb | | | |

Journal of Cell Science • Supplementary information

**Table S4. Secondary antibodies used in this study.**

| Antibody | Manufacturer (catalogue numer) | Host | Antigen | Dilution |
|---|---|---|---|---|
| **6nm Gold** | Jackson ImmunoResearch Laboratories (706-195-148) | Donkey | Guinea pig IgG | 1:10 |
| **6nm Gold** | Jackson ImmunoResearch Laboratories (111-195-144) | Goat | Rabbit IgG | 1:10 |
| **HRP** | Jackson ImmunoResearch Laboratories (106-035-003) | Goat | Guinea pig IgG | 1:10,000 |
| **HRP** | Jackson ImmunoResearch Laboratories (706-035-148) | Donkey | Guinea pig IgG Cross-adsorbed | 1:10,000 |
| **HRP** | Jackson ImmunoResearch Laboratories (111-035-003) | Goat | Rabbit IgG | 1:10,000 |
| **HRP** | Jackson ImmunoResearch Laboratories (711-035-152) | Donkey | Rabbit IgG Cross-adsorbed | 1:25,000 |
| **HRP** | Jackson ImmunoResearch Laboratories (115-035-003) | Goat | Mouse IgG | 1:10,000 |
| **AlexaFluor™ 488** | Thermo Fisher Scientific (A-11008) | Goat | Rabbit IgG | 1:200 |
| **AlexaFluor™ 488** | Thermo Fisher Scientific (A-10680) | Goat | Mouse IgG | 1:300 |
| **Texas Red** | Jackson ImmunoResearch Laboratories (115-075-044) | Goat | Mouse IgG | 1:150 |
| **Texas Red** | Jackson ImmunoResearch Laboratories (106-075-003) | Goat | Guinea pig IgG | 1:50 |

Journal of Cell Science • Supplementary information
